# Genomic Characterisation of Mushroom Pathogenic Pseudomonads and Their Interaction with Bacteriophages

**DOI:** 10.3390/v12111286

**Published:** 2020-11-10

**Authors:** Nathaniel Storey, Mojgan Rabiey, Benjamin W. Neuman, Robert W. Jackson, Geraldine Mulley

**Affiliations:** 1 School of Biological Sciences, Whiteknights Campus, University of Reading, Reading RG6 6AJ, UK; nathaniel_storey@hotmail.com (N.S.); r.w.jackson@bham.ac.uk (R.W.J.); g.mulley@reading.ac.uk (G.M.); 2 School of Biosciences and Birmingham Institute of Forest Research, University of Birmingham, Birmingham B15 2TT, UK; 3 Biology Department, College of Arts, Sciences and Education, TAMUT, Texarkana, TX 75503, USA; bneuman@tamut.edu

**Keywords:** bacteriophage, mushroom diseases, *Pseudomonas*, *Agaricus*, genome, genetic interaction

## Abstract

Bacterial diseases of the edible white button mushroom *Agaricus bisporus* caused by *Pseudomonas* species cause a reduction in crop yield, resulting in considerable economic loss. We examined bacterial pathogens of mushrooms and bacteriophages that target them to understand the disease and opportunities for control. The *Pseudomonas* *tolaasii* genome encoded a single type III protein secretion system (T3SS), but contained the largest number of non-ribosomal peptide synthase (NRPS) genes, multimodular enzymes that can play a role in pathogenicity, including a putative tolaasin-producing gene cluster, a toxin causing blotch disease symptom. However, *Pseudomonas* *agarici* encoded the lowest number of NRPS and three putative T3SS while non-pathogenic *Pseudomonas* sp. NS1 had intermediate numbers. Potential bacteriophage resistance mechanisms were identified in all three strains, but only *P. agarici* NCPPB 2472 was observed to have a single Type I-F CRISPR/Cas system predicted to be involved in phage resistance. Three novel bacteriophages, NV1, ϕNV3, and NV6, were isolated from environmental samples. Bacteriophage NV1 and ϕNV3 had a narrow host range for specific mushroom pathogens, whereas phage NV6 was able to infect both mushroom pathogens. ϕNV3 and NV6 genomes were almost identical and differentiated within their T7-like tail fiber protein, indicating this is likely the major host specificity determinant. Our findings provide the foundations for future comparative analyses to study mushroom disease and phage resistance.

## 1. Introduction

*Agaricus bisporus* is the most commonly cultivated mushroom in Europe and North America, most frequently known as the “button mushroom” or “Portobello mushroom”. Global production of *A. bisporus* is reported to exceed 1.8 × 10^6^ tonnes per year, of which 8–9 × 10^5^ tonnes are cultivated in Europe alone, worth an approximate 2 billion Euros annually [1]. Several pathogenic pseudomonads are known to cause discolouration of *A. bisporus*, termed “blotch disease”, including *Pseudomonas tolaasii*, that causes brown blotch [2] and *P. agarici*, that causes both yellow blotch and a disease of the mushroom gill structures called drippy gill [3]. The disease leads to reduced quality [4,5], which cause losses of up to 25% of the total mushrooms grown, with some further 10% downgraded in market value [6,7].

A variety of different *Pseudomonas* bacteria can cause disease symptoms in mushrooms [8], including *Pseudomonas gingeri* [9], *Pseudomonas fluorescens* and *Pseudomonas putida* [10], *Pseudomonas protegens* [11], *Pseudomonas* NZI7 [12] and *Pseudomonas constantinii* [13]. The best characterised strains are *P. tolaasii* and *P. agarici*, two species known to cause discolouration and blotch disease and drippy gill disease, respectively, in the edible white mushroom, *A. bisporus*. *P*. *tolaasii* has also been reported to infect *Arabidopsis* cotyledons and cause chlorosis and growth arrest of the seedling, indicating there may be some overlapping virulence mechanisms [14]. There have been limited studies examining the genetic and mechanistic basis for how these pathogens cause disease in different niches of the mushroom and how these pathogens might be controlled. Most disease studies have worked on the small peptide toxin, tolaasin [14,15], while there have been various management strategies and control regimes [8] including some studies on biocontrol using bacteriophages [16,17].

*P. tolaasii* causes small brown or cream lesions on the pileus and stipe that become darker and more sunken as the damage progresses, and the individual lesions may merge to cover the entire surface of the mushroom [1]. Phylogenetic analysis based on 16S rDNA places *P. tolaasii* in the *Pseudomonas fluorescens* subgroup [18] and has been officially placed in the taxonomic group of fluorescent *Pseudomonas* biotype II [1]. *P. tolaasii* can undergo phenotypic variation, a tactic that is beneficial for its survival in nutrient-poor environments [19].

*P. agarici* is the causal organism of “drippy gill”, typified by small, dark pigmented spots on the gills of mature mushrooms that contain a light cream or grey spot in their centre [20]. The infection of the gills may cause a collapse of the locally affected tissue or in the case of severe infections the bacterial droplets may coalesce between gills and lead to a total collapse of gill tissue [21]. Phylogenetic analysis based on 16S rRNA gene topology analysis places *P. agarici* in the *P. fluorescens* intragenic cluster [21,22]. However, further phylogenetic analysis of *P. agarici* involving four “housekeeping” genes 16S rRNA, *gyrB*, *rpoB,* and *rpoD* failed to place *P. agarici* in any specific *Pseudomonas* subgroup [23].

The *Pseudomonas* genus is well known for its production and use of secondary metabolites which play roles in pathogenicity on a range of different hosts. They have been reported to produce compounds such as lipids, phenazines, pyrroles, indoles, amino acids and peptides, pterines, and other miscellaneous compounds that play important roles in virulence and pathogenicity [24]. One method for production of peptide secondary metabolites in pseudomonads is via the use of non-ribosomal peptide synthetases (NRPSs), which are very large multimodular enzymes that synthesise specific peptide products. NRPSs are comprised of multiple domains which each contain three catalytic domains [25]. The NRPS produced secondary metabolites perform a wide variety of roles such as in phytopathogenicity, microbial and predatory antagonism, swarming motility, and biofilm formation [26,27]. In terms of mushroom diseases, little is known about the virulence factors employed by bacteria that lead to disease symptoms. The soft rot disease of *A. bisporus*, caused by *Burkholderia gladioli pv. agaricicola* (formerly known as *Pseudomonas cepacia* and *Pseudomonas gladioli pv. agaricicola)*, is caused by virulence factors including degradative enzymes such as chitinase and protease that are secreted by the Type II secretion system [28]. *Janthinobacterium agaricidamnosum* causes pitting and sticky blotches on *A. bisporus* cap and these symptoms are caused by a singular virulence factor, jagaricin, which is produced as a secondary metabolite by NRPS [29]. *P. costantinii* and *P. tolaasii* both cause brown blotch disease and produce tolaasin, a secreted toxin produced by NRPS, which replicates symptoms of blotch disease on mushrooms [30,31,32].

Current control measures to lower mushroom pathogen spread have included the use of chemicals, host environmental management, and biocontrol agents (plant extracts and antagonistic microbes) [33,34,35,36]; however, none are fully efficient in managing the disease [37]. Chemical controls are increasingly becoming less popular due to mounting concerns over the adverse effects of chemicals on both the environment and consumer health. Phenotypic plasticity of *Pseudomonas* species to tolerate adverse environmental conditions makes disease control very challenging [38,39,40] and can result in huge crop losses during severe epidemics [37].

One potential alternative to control bacterial diseases is through bacteriophage biocontrol. Bacteriophages (phages) are viruses that infect bacteria, using the bacterial transcription and translation machinery to complete their replication cycle, redirecting, and hijacking of host metabolism, and ultimately resulting in the lysis of the host bacterial cell. Their ability to persist and proliferate in the environment and high degree of host specificity make phages an attractive biocontrol agent for *Pseudomonas* infections of cultivated mushrooms [41,42]. However, little research has been conducted on phage capable of infecting mushroom pathogens *P*. *agarici* and only a small number of phages have been characterised that infect *P*. *tolaasii* [43,44]. Kim, et al. [43] isolated environmental phages that were capable of suppressing blotch formation on oyster mushroom (*Pleurotus ostreatus*) surfaces when applied simultaneously with *P. tolaasii.*

During host infection, a bacteriophage attaches to a bacterium and injects its genetic material (DNA or RNA) into the cell. The host cell machinery is then used to copy the phage genome and translate/express the proteins used for making the virus structural components. Most dsDNA phage use a lytic enzyme, a lysin or endolysin, to achieve lysis of their hosts and release progeny virions into the external environment to infect neighbouring cells. The host cell lysis system of a bacteriophage, also termed the “lysis cassette”, can be broadly categorised into two functional classes: canonical and signal-anchor-release (SAR) lysis systems. Both systems require the action of transmembrane proteins called holins to control the precise timing of host cell lysis; however, the mechanisms by which these proteins perform their roles differs dramatically between the two classes [45,46].

The host bacteria can evolve resistance to bacteriophage using different mechanisms [47,48]. This can be via the modification of the cell surface, such as receptor mutation or receptor masking, injection blocking, superinfection exclusion systems encoded by resident prophages, BREX (bacteriophage exclusion), restriction modification or abortive infection [47,49,50,51,52]. The CRISPR (clustered regularly-interspaced palindromic repeats) is a potential mechanism used by bacteria in defence system functions against phage infections. This involves the identification of the phage genome via the Cas9 endonuclease, subsequently incorporating part of the phage genome into the bacterial genome. This then produces a nucleic acid construct from the incorporated phage genome that can bind to other phage with the same genomic sequence, leading to phage inactivation [53].

In this study we focused on two mushroom pathogens, *P. tolaasii* and *P. agarici*, to identify potential modes of virulence and phage resistance mechanisms. We also isolated phages that infect the bacteria and sequenced three phage genomes to gain insight to potential cell lysis and host specificity determinants, which are important for identifying phages that can be used safely and consistently in disease control.

## 2. Materials and Methods

### 2.1. Bacterial Strains and Culture Media

*P. tolaasii* NCPPB 2192T and *P. agarici* NCPPB 2472 were used as host strains for isolation and propagation of phage isolates. Kings Medium B (KB, 1 L distilled H_2_O, 20 g protease peptone, 1.5 g K_2_HPO_4_, 1.5 g MgSO_4_.7H_2_O, 10 mL glycerol (plus 15 g L^−1^ agar for plates) [54] and Lysogeny Broth (LB, tryptone 10 g L^−1^; yeast extract 5 g L^−1^; NaCl 10 g L^−1^ ) broth [55] were used for culturing the bacterial strains. KB with 0.7% agar was used in the soft top agar overlay for the plaque assays. Phosphate buffered saline (PBS, NaCl 8 g L^−^^1^; KCl 0.2 g L^−1^; Na_2_HPO_4_ 1.44 g L^−^^1^; KH_2_PO_4_ 0.24 g L^−^^1^) was used to prepare phage dilutions.

### 2.2. Pathogenicity Test

To develop a suitable pathogenicity model, the bacteria *P. tolaasii* NCPPB 2192T and *P. agarici* NCPPB 2472 were examined for their ability to cause the symptoms of disease associated with brown blotch and drippy gill, respectively, on *Agaricus bisporus* mushrooms. A series of inoculations were performed on excised “button” (immature, unopened) mushroom caps sourced from a local supermarket, which showed no prior signs of browning or lesions. A single colony of either *P. tolaasii* NCPPB 2192T or *P. agarici* NCPPB 2472 was taken from a streak plate on KB agar after 24 h and used to inoculate 10 mL of KB, before overnight incubation at 27 °C with shaking. This overnight culture was then pelleted at 1500 g for 30 min and the pellet resuspended in 2 mL PBS. A 10 μL aliquot of the bacterial suspension (≃10^6^ colony forming unit (cfu) per mL) was then applied to either the mushroom cap for *P. tolaasii* or for *P. agarici* the mushroom hymenial tissue was removed and the suspension applied to the exposed gill tissue. Care was taken to avoid damaging the tissue during pipetting. The inoculated mushrooms were then placed into sterile beakers which were sealed with cling-film and incubated at 27 °C until disease symptoms appeared.

### 2.3. Phage Sampling and Isolation

Water samples were taken from the River Thames (51°29′07.3″ N 0°07′38.2″ W) and untreated sewage (51°27′46.0″ N 0°12′05.4″ W). A 10 mL volume of water samples was combined with an equal volume of overnight culture of either *P. tolaasii* NCPPB 2192T or *P. agarici* NCPPB 2472, followed by overnight incubation at 27 °C with 220 rpm shaking. The bacteria were pelleted by centrifugation at 1500× *g* and the supernatant was removed and filtered through a 0.45 μm filter (Millipore, Sigma-Aldrich, Gillingham, UK) and stored at 4 °C.

For phage isolation 100 μL of the supernatant from each sample was serially diluted in PBS and was then plated on KB agar plates and overlaid with the soft top agar containing 100 μL of either *P. agarici* NCPPB 2472 or *P. tolaasii* NCPPB 2192T and the filtered water sample. The plates were then incubated for 24–48 h at 27 °C and observed for the presence of visible plaques. If visible plaques were present, a new overnight culture of either *P. tolaasii* or *P. agarici* was inoculated with a stab of an isolated phage plaque before being incubated overnight at 27 °C. The overnight culture was then centrifuged at 1500× *g* for 30 min to pellet cells and the supernatant filtered through a 0.45 μm filter and 100 μL of filtered diluted supernatant plated on KB agar plates overlaid with the soft top agar containing either *P. agarici* or *P. tolaasii* before incubation at 27 °C overnight. The plates were then checked for uniform plaque morphology and if there was variable plaque morphology the process was repeated with each distinct plaque morphology until a uniform morphology was attained for each inoculation. Phages were amplified by plating 10^6^ plaque forming units (pfu) per plate with bacterial host on a soft agar overlay plate and incubated overnight at 27 °C. Then, 5 mL PBS was added onto the plate and incubated at room temperature for 90 min with agitation every 15 min. PBS was removed and filtered through a 0.45 µm filter to remove any bacteria.

### 2.4. Phage Host Range Assay

Phage host range assays were performed using 100 µL of 10^6^ pfu ml^−1^ phage on plates with a soft agar layer containing 100 µ of *Pseudomonas* species, *P. tolaasii* NCPPB 2192T, *P. agarici* NCPPB 2472, *Pseudomonas* sp. NS1, *P. syringae pv. morsprunorum* R1 5244, *P. syringae pv. syringae* 9097, *P. syringae pv. morsprunorum* R2 5255, *P. fluorescens* PfO-1, and *P. aeruginosa* 14207. The plates were incubated at 27 °C for 24 h. After incubation, plates were checked for clearing where phage were applied.

### 2.5. Electron Microscopy

To visualise the phage using transmission electron microscopy (TEM), copper coated carbon-formvar grids (300 mesh, Agaro) were prepared via floating on 10 µL drops of filtered phage lysate solutions of roughly 10^6–8^ pfu mL^−1^ for 10 min. This was negatively stained for 2 min with 10 μL of 1% *w/v* uranyl acetate (ThermoFisher Scientific, Loughborough, UK) dissolved in nanopure water. Samples were analysed using a Phillips CM200 TEM at 80 V and photos taken using AMT camera system software.

### 2.6. Experimental Coevolution

The coevolutionary assays, to determine the patterns of coevolution between phages and bacteria, were performed using a method adapted from Betts, et al. [56]. The initial phage–host incubation was performed in 6 mL of KB in a 30 mL tube with 5 μL of ϕNV3 phage (~5 × 10^5^ pfu) and with 10 μL of *P. agarici* (~2 × 10^6^ cfu), which was incubated at 27 °C for 24 h (MOI ~0.25). From this overnight phage–bacteria culture 100 μL was taken and transferred to a new tube containing 6 mL of fresh KB and incubated for 24 h at 27 °C. This process was repeated for a total of 12 sequential transfers (T). For each of the transfers 1 mL of bacteria–phage mixture was removed and stored in 40% glycerol at −80 °C; phage was isolated from each transfer by adding 10% *v/v* chloroform to bacteria/phage culture, mixed by vortexing for several seconds followed by centrifugation for 4 min at 10,000× *g* before the supernatant of purified phage was removed and stored at −80 °C. Phage infection capacity for bacterial transfers T1, T3, T5, T7, and T9 was measured by streaking of 16 arbitrarily sampled bacterial colonies from streak plates of each transfer that were incubated at 27 °C for 72 h on KB agar. Each bacterial transfer was tested against the past, present, and future phage transfers (T − 1, T0, T + 1). To each of the 16 arbitrarily sampled bacterial streaks 5 μL of purified phage was placed in the centre of each line. This process was repeated 5 times per phage time point (past, present and future). After incubation for 72 h at 27 °C a bacterial colony was considered resistant if its growth continued uninterrupted by the addition of phage.

### 2.7. Genome Sequencing

*P. agarici* NCPPB 2472, *P. tolaasii* NCPPB 2192T, and *Pseudomonas sp.* NS1 total genomic DNA was isolated and purified using a Sigma-Aldrich GenElute™ Bacterial Genomic DNA Kit following the manufacture’s instruction. A Phage DNA Isolation Kit (Norgen Biotek, Thorold, Ontario, Canada) was used to extract phage DNA from a high titer plate lysate (minimum of 10^8^ pfu mL^−1^) following the manufacturer’s instructions. Extracted DNA was stored in a 1.5 mL ultracentrifuge tube at −20 °C until needed. The DNA concentration was measured using a Nanodrop 2000 (ThermoFisher Scientific, Loughborough, UK).

De novo paired-end sequencing was performed by Source Bioscience (Nottingham, UK) on a single lane of an Illumina MiSeq with a read length of 50 bp. Whole genome sequencing libraries were generated by an Illumina TruSeq DNA Nano kit. Data was adapter and quality trimmed using Skewer v0.1 [57] and trimmed reads were assembled into contigs with SPAdes v3.6.2 [58].

Mapping of *P. agarici* NCPPB 2472 contigs was performed using both *P. fluorescens* A506 (GCA_000262325.2) and *P. agarici* 2289 (GCA_000280785.1) as reference sequences using CONTIGuator 2.7.4 [58]. Mapping of the *P. tolaasii* NCPPB 2192T contigs was performed using *P. tolaasii* PMS117 as a reference genome using MeDuSa v1.6 [59]. Mapping of *Pseudomonas sp.* NS1 contigs was performed using *Pseudomonas azotoformans* S4 (GCA_001579805.1), as a reference sequence using CONTIGuator 2.7.4 [60].

### 2.8. Secondary Metabolites

Putative biosynthetic gene clusters were predicted from the full genomic sequence files of *P. tolaasii*, *P. agarici,* and *Pseudomonas sp.* NS1 using antiSMASH 3.0 [61]. These putative clusters were then checked manually using both BLASTn and BLASTp and checked for predicted function. To identify open reading frames (ORFs) potentially encoding large NRPS’s, all ORFs over 1000 amino acids (aa) in length were identified and analysed with BLASTp. ORFs identified as potentially encoding NRPSs were then analysed using InterPro v65.0 [62] for the presence of condensation, adenylation and thiolation domains.

### 2.9. Type III, Type IV and Type VI Secretion Systems

The protein secretion system gene clusters were predicted from the full genomic sequence of *P. tolaasii*, *P. agarici,* and *Pseudomonas sp.* NS1 using KofamKOALA [63] and manual searches of annotations in Artemis [64]. These putative clusters were then checked manually using both BLASTn and BLASTp and checked for predicted function. tRNAscan-SE was used to search for tRNA genes within the genome sequences [65].

### 2.10. Endolysin Constructs

To allow for expression of the NV1 cell lysis proteins, two constructs were created using primers (Table S1) containing the native stop codon and the vector pEXP5-CT/TOPO^®^ (Invitrogen, ThermoFisher Scientific, Loughborough, UK). PCR amplification was performed using colony PCR and the product checked by agarose gel electrophoresis. The band corresponding to the desired product was confirmed by sequencing using the T7 primers. The products were TOPO^®^ cloned into the pEXP5-CT/TOPO^®^ vector as per the manufacturer’s protocol (Invitrogen). Following the cloning reaction, the products were transformed into One Shot^®^ TOP10 Chemically Competent *Escherichia coli*, following the manufacturer’s protocol (Invitrogen), and plated onto selective LB agar plates [55] containing ampicillin (100 μg mL^−1^), then incubated at 37 °C overnight. Colonies were checked for the presence of the respective NV1 holin and lysozyme genes via colony PCR and sequenced for confirmation. The colonies which contained the required inserts were then grown in LB broth containing ampicillin (100 μg mL^−1^) and incubated for 24 h at 37 °C. The constructs were then purified from the cultures using a QIAprep Spin Miniprep kit (Qiagen, Manchester, UK). The sequence of the products was then confirmed by Sanger sequencing (Source Bioscience, Nottingham, UK) with T7 primers. The method for producing the constructs for the ϕNV3 lysis proteins was the same as for the NV1 constructs. Each of the lysis proteins was cloned separately into pEXP5-CT/TOPO^®^ (pinholin, lysozyme, Rz-like protein), as well as one construct containing both the pinholin and lysozyme and one construct containing all the lysis proteins (Table S3).

### 2.11. Protein Expression

Expression of the phage lysis proteins was performed by first transforming lysis protein constructs into BL21-AI™ One Shot^®^ Chemically Competent *E. coli* using the manufacturers protocol (Invitrogen); all growth media was supplemented with 0.1% (*w/v*) glucose to prevent unintentional expression due to the toxic nature of the recombinant proteins. Following incubation of the transformation plates at 37 °C overnight, colonies were picked from the plates and cultured overnight at 37 °C in 10 mL LB supplemented with 0.1% glucose and ampicillin and analysed by PCR using the appropriate primers (Tables S1 and S2) corresponding to the relevant lysis proteins and confirmed by Sanger sequencing (Source Bioscience, Nottingham, UK) with T7 primers. For the protein expression assay, 1 mL of BL21-AI cells containing phage lysis protein sequences was pipetted into 10 mL of 0.1% glucose supplemented with ampicillin LB broth and incubated at 37 °C with 220 rpm shaking until the optical density (OD_600_) reached approximately 0.4 (≃10^8^ cfu per mL). 100 μL of each culture was then pipetted into a Cellstar^®^ 96 well cell culture plate. To 6 of each of the culture containing wells, 2 μL of 10% L-Arabinose solution (Sigma-Aldrich, Gillingham, UK) was added to induce protein expression and the plate immediately transferred to a Tecan GENios microplate reader pre-warmed to 37 °C and maintained at 37 °C while OD_595_ readings were taken every 5 min for 150 cycles with rotary shaking between readings.

## 3. Results

### 3.1. Pathogenicity Test

We first aimed to test that the two mushroom pathogen strains, obtained from an external culture collection, were capable of causing disease on mushrooms. The pathogenicity test revealed that after 72 h, the mushrooms infected with *P. tolaasii* NCPPB 2192T had begun to display darkening of the tissue and pitting of the surface, confirming the ability of the strain to cause brown blotch disease. *P. agarici* NCPPB 2472 produced cloudy-white bacterial droplets on the mushroom gill tissue after 72 h of incubation at 27 °C. This confirmed its ability in infecting and causing drippy gill disease symptoms on *A.s bisporus* mushroom gill tissue.

During an experiment to enumerate *P. tolaasii* from the surface wash of *A. bisporus* mushrooms showing no symptoms of disease, a different colony morphology was identified on culture plates in addition to that expected for *P. tolaasii*. It was hypothesised from the colony morphology and Gram stain results that the strain may be a member of the fluorescent pseudomonads or potentially a faster growing strain of *P. tolaasii*. It was initially identified as a *Pseudomonas* and later confirmed by genome sequencing and named “*Pseudomonas* sp. NS1”. *Pseudomonas* sp. NS1 was unable to cause any disease symptoms on the surface of *A. bisporus* cap after inoculation.

### 3.2. Bacterial Whole Genome Sequencing

To investigate how closely related the bacteria used and isolated in this study are, the potential mechanisms of mushroom pathogenicity and virulence, and how they might resist phage infection, genomic DNA from the two mushroom pathogens and *Pseudomonas* sp. NS1 was extracted, and genome sequencing performed (Table 1). The *P. tolaasii* NCPPB 2192T genome (GenBank accession number: CP020369.1) was 6,856,683 bp in length with 6286 putative ORFs, of which 6065 were identified as being protein coding sequences, and 63 RNA encoding genes were identified including 56 tRNAs, 3 complete rRNAs and 4 ncRNAs.

The *P. agarici* NCPPB 2472 genome (GenBank accession number: CP014135.1) was substantially smaller at 5,618,560 bp in length. The genome was mapped to two related *Pseudomonas* strains, *Pseudomonas fluorescens* A506 and *Pseudomonas agarici* NCPPB 2289. Of the 4901 ORFs identified, 4673 were identified as being protein coding and 67 were RNA encoding genes, including 59 tRNAs, 4 complete rRNAs and 4 ncRNAs.

*Pseudomonas* sp. NS1 (GenBank accession number: CP022960) sequence was 6,702,516 bp in length. The genome was mapped to the genome of *Pseudomonas azotoformans* S4 (GenBank accession number: CP014546). Of the 6241 ORFs identified, 6,073 were identified as being protein coding and 72 were identified as RNA encoding genes, including 60 tRNAs, 8 complete rRNAs, and 4 ncRNAs.

To further aid in taxonomic classification and to calculate the probability that *Pseudomonas* sp. NS1 was a strain of either *P. azotoformans* or *P. fluorescens* the full draft genome was analysed using the JSpecies [65] work package. The results of the pairwise comparison of the draft genome of *Pseudomonas* sp. NS1 showed that it was most closely related to *P. fluorescens* LMG 5329 (GenBank Accession number: ASGY00000000.1) and *P. azotoformans* S4 with similarity of 93.43% and 93.32%, respectively. The genome sequences were used to identify key virulence determinants for the two pathogens in comparison to *Pseudomonas* sp. NS1.

Gene locus tags are indicated as: B5P22_RSXXXXX (*P. tolaasii* NCPPB 2192T), AWM79_RSXXXXX (*P. agarici* NCPPB 2472) and CI807_RSXXXXX (*Pseudomonas* sp. NS1), but for simplicity locus tags are only shown as RSXXXXX throughout the bacterial genome analysis.

#### 3.2.1. Secondary Metabolites and NRPSs

*P. tolaasii* NCPPB 2192T

Screening of the genome sequence with antiSmash to find potential biosynthetic gene clusters revealed a total of 17 complete and incomplete putative NRPS encoding ORFs, ranged from 675 bp to 21,173 bp in length. NRPS products have been identified as virulence factors. Five putative NRPSs (RS30620, RS22935, RS23980, RS02650, and RS24725) were identified that showed both amino acid and nucleotide similarity to a biosynthetic cluster from *P. costantinii* (causal agent of brown blotch disease), which encodes tolaasin, a secreted toxin that causes symptoms of blotch disease of mushrooms. ORF RS02650 was predicted to have 82% and 77% nucleotide similarity to the *P. costantinii taaC* and *taaD* gene and associated protein products, respectively.

The production of siderophores, including pyoverdine, plays roles in competition and virulence in *Pseudomonas* species. Two of the NRPSs in the *P. tolaasii* NCPPB 2192T genome, RS16165 and RS16170 (1527 aa and 224 aa in size, respectively) had 57% identity to pyoverdine synthesis genes PvdI of *P. syringae pv. daphniphylli* (causal agent of bacterial gall of cherry trees, GeneBank accession: KPX09284.1) and 68% identity to the pyoverdine sidechain peptide synthetase of a *P. fluorescens* strain (GenBank accession: WP_123448596.1). These genes were also found to be upstream of a further incomplete NRPS, RS16160, that showed 63% amino acid identity to pyoverdine synthesis protein PvdJ of *P. aeruginosa* strain 62 (GeneBank accession: ERX81199.1) and downstream of a TonB-dependent siderophore receptor (RS16175). Similarly, a 1948 aa NRPS was identified (RS12420) that is also hypothesised to be involved in siderophore synthesis, due to its location downstream of a gene predicted to encode a siderophore synthetase (RS12390), MFS transporter (RS12395) and TonB-dependent receptor (RS12400); as well as being located upstream from a TonB-dependent siderophore receptor (RS12430) (Table S4).

*P. agarici* NCPPB 2472

Screening of the genome sequence with antiSmash to find potential biosynthetic gene clusters revealed two NRPS clusters; the first cluster compromised of 5 NRPS proteins (RS12910, RS12915, RS12920, RS12925, and RS00065). The second was comprised of a single NRPS (RS20800) and a polyketide synthase (RS20810); the final biosynthetic cluster appears to possess only a single NRPS (RS10630). While all the NRPSs showed a high degree of conservation with other NRPSs of the *Pseudomonas*, it was not possible to predict the function of these biosynthetic clusters from *in silico* analysis.

AntiSmash identified a biosynthetic cluster (RS04660 to RS04705) that is predicted to produce the siderophore achromobactin. Three proteins (RS04680, RS04695, and RS04705) showed high amino acid identity to the proteins AcsD, AcsC, and AcsA, respectively, which have been demonstrated to be involved in the biosynthesis of achromobactin by *P. syringae pv. syringae* B728a. A fourth protein (RS04685), which was predicted to function as a diaminopimelate decarboxylase, might perform the function of the *P. syringae pv. syringae* B728a protein AcsE. The gene cluster also showed significant similarities to the achromobactin biosynthesis cluster of *P. syringae pv. syringae* B728a. All ORFs showed 72–91% amino acid identity to the corresponding ORFSs of *P. syringae pv. syringae* B728a. The predicted function of this cluster in the production of a siderophore is further confirmed by the presence of a predicted TonB-dependent siderophore receptor (RS04670) and predicted siderophore biosynthesis protein SbnG (RS04700) (Table S4).

Pseudomonas sp. NS1

In total 10 complete or partial NRPSs were identified within the genome of *Pseudomonas* sp. NS1, with 8 of these located in two distinct clusters. A NRPS-containing biosynthetic gene cluster (RS19185 to _19300) containing six whole and partial NRPSs was predicted by antiSMASH to potentially produce the lipopeptide poaeamide. However, BLASTn analysis of the NRPSs within the cluster showed that it had 84% nucleotide identity to the lipopeptide production system gene cluster for white line-inducing principle (WLIP) of *Pseudomonas fluorescens* strain LMG 5329 cluster 2. This would indicate that it is more likely this gene cluster encodes the WLIP, a lipopeptide of the viscosin group, than poaeamide.

A second NRPS (RS26350) showed 94% nucleotide identity to the WLIP production cluster 1 of *P. fluorescens* LMG 5329. Further in silico analysis showed that these NRPSs, not only showed nucleotide identity, but also conserved synteny of genes when compared to the WLIP production cluster 2 of *P. fluorescens* strain LMG 5329. To determine if *Pseudomonas* sp. NS1 could produce WLIP, it was cultured alongside *P. tolaasii* NCPPB 2192T. A distinct white precipitate formed within the agar, which indicates the biosynthetic gene clusters identified most likely encode the NRPS system for WLIP production rather than any other of the viscosin-related nonapeptides (Supplementary Materials Table S4).

#### 3.2.2. Type III, Type IV, and Type VI Secretion Systems

In terms of pathogenicity, several protein secretion systems can move effector proteins out of the bacterial cell to the millieu or inside the target cells and thus represent major virulence determinants for bacteria harbouring them. The major secretion systems that can translocate effector proteins are Type III (T3SS), Type IV (T4SS), and Type VI (T6SS) secretion systems. These effectors produce physiological changes in the host, fulfilling essential functions during the interaction between bacteria and eukaryotes. We therefore examined the genome sequences for these gene systems and found evidence for all three systems existing either fully or partially in the genomes (see (Results and Table S5)).

#### 3.2.3. Identification of Phage-Related Sequences and Putative Phage Resistance Systems in the Bacterial Genomes

##### Prophage Sequences

*P. tolaasii* NCPPB 2192T

Screening of the genome sequence with PHASTER [66] identified nine prophage regions, of which five regions were intact, three regions were incomplete, and one region was questionable. The intact regions were identified to have similarity to other phages; the first intact region (position 496286–529725 bp, 33.5 kb) had 70% similarity to *Pseudomonas* phage JBD69 (NC_030908.1), the second intact region (position 4609424-4661540 bp, 52.1 kb) had 70% similarity to *Burkholderia* phage Bcep176 (NC_007497.1); these two regions had one Cro/Cl family transcriptional regulator. The third region (position 4,901,329-4,933,507 bp, 32.1 kb) had 69% similarity to *Salmonella* phage 118970_sal3 (NC_031940.1) and the fourth region (position 5295871-5339955 bp, 44 kb) had 75% similarity to *Pseudomonas* phage vB_PaeP_PPA-ABTNL (NC_027375.1), and fifth region (position 6763248-6817888 bp, 54.6 kb) had 97% similarity to *Pseudomonas* phage UFV-P2 (NC_018850.2) (Table S6).

*P. agarici* NCPPB 2472

PHASTER identified two incomplete prophage regions (Table S6).

Pseudomonas sp. NS1

PHASTER identified five prophage regions, of which one region was predicted to be intact and four regions were incomplete. The intact region (position 3132347-3178632 bp, 46.2 kb) had 68% similarity to *Escherichia* phage vB_EcoM_ECO1230-10 (NC_027995.1) (Table S6).

##### Phage Tail-Like Bacteriocin

*P. tolaasii* NCPPB 2192T

Phage tail-like bacteriocins (PTLBs) are high-molecular-weight bactericidal protein produced by bacteria that kill other types of bacteria. They are evolutionarily related to the tail proteins of various bacteriophages tail structures, such as type VI secretion systems, which target insect cells, or phage tail-like “arrays” involved in interactions between bacteria and eukaryote hosts [67]. PTLBs can be grouped into two types. The F-type are phylogenetically related to the tails of *Siphoviridae* bacteriophage and are non-contractile, comprised only of a tube and no sheath. The R-type PTLBs are related to the tails of *Myoviridae* phage and are contractile, consisting of a central tube surrounded by a sheath, which is connected to an additional baseplate structure to which receptor-binding proteins such as tail fibers are bound. Some bacteria, particularly *P. aeruginosa*, produce both R- and F-type bacteriocins. PTLBs first bind to a target cell via RBPs and once bound to the target, PTLBs cause rapid death, through membrane depolarisation in a single hit mechanism.

A biosynthetic gene cluster comprised of 11 genes (RS16310 to RS16360) was identified in the genome of *P. tolaasii* NCPPB 2192T, hypothesised to be involved in the synthesis of PTLB. Downstream of the predicted PTLB gene cluster, a further protein (RS16305) was identified, with 100% amino acid identity to the repressor protein PrtR of *P. fluorescens* strain PCL1751 and is therefore likely to be involved in transcriptional regulation of the identified PTLB in tandem with another protein, the pyocin activator protein PrtN (RS06590) (Table S7).

*P. agarici* NCPPB 2472

A biosynthetic gene cluster hypothesised to be involved in the synthesis of a phage tail-like bacteriocin was identified (RS10925 to _11005). Similar to the PTLB cluster identified in *P. tolaasii* 2192T an XRE family transcriptional regulator was identified downstream of the PTLB gene cluster (RS10920), which may function in a role similar to the PrtR protein. An incomplete gene, predicted to encode the pyocin activator protein PrtN (RS18570), was also discovered but it is not clear whether this may retain functionality for transcriptional regulation of the PTLB cluster (Table S7).

#### 3.2.4. Phage Resistance Systems

*P. tolaasii* NCPPB 2192T

CRISPR is a potential mechanism used by bacteria in defending against phage infections, but no CRISPR-associated repeats or proteins were identified in the *P. tolaasii* NCPPB 2192T genome.

However, multiple restriction endonuclease proteins were identified within the genome of *P. tolaasii* NCPPB 2192T. The predicted “restriction endonuclease” (RS17360) contains an McrB conserved domain, which is associated with the GTP-binding regulatory subunit. The RS17360 predicted restriction endonuclease gene is upstream of a “hypothetical protein” (RS17355) that contains a PDDEXK_7 conserved domain, which is a domain of the PD-(D/E)XK nuclease family and could function as a methylation-dependent restriction enzyme; therefore, it is likely that both RS17360 and RS17355 are two subunits of a single uncharacterised Type IV methylation-dependent restriction endonuclease system. A second predicted restriction endonuclease (RS17410) is predicted to be a Type III restriction endonuclease and is directly downstream of a site-specific DNA-methyltransferase (RS17420), with a second site-specific DNA-methyltransferase (RS17440) approximately 7700 bp upstream of the first. The incomplete methyltransferase encoded by RS26390, predicted as a “restriction endonuclease subunit M” shows significant amino acid identity to a known prophage (Prophage PSPPH02) adenine modification methyltransferase, and therefore has no associated restriction endonuclease. Likewise, the incomplete methyltransferases encoded by RS30090 and RS30095 are likely to be of phage origin; the protein encoded by RS30090 shows 100% amino acid identity to a protein of the prophage PSPPHO2 (by BLASTp) and the protein encoded by RS30095 shows a high degree of similarity to prophage PSPPH06 (95% amino acid similarity (by BLASTp)). Furthermore, there are several ORFs downstream of both these genes that encode phage related proteins, including RS30070 (baseplate assembly protein) and RS30080 (microvirus H family protein).

Alginate biosynthesis has been extensively studied in *Pseudomonas aeruginosa*, where it functions as a major virulence factor and possibly acts to mask cell surface receptors from phage. A biosynthetic operon comprised of 15 genes (RS11470 to RS11545) was identified in the genome of *P. tolaasii* NCPPB 2192T, hypothesised to be involved in the production of alginate due to the presence of a gene encoding the alginate biosynthesis protein Alg44 (Table S8).

*P. agarici* NCPPB 2472

The genome of *P. agarici* NCPPB 2472 was found to contain a single CRISPR-associated repeat region, between 1180063-1181413 bp (RS05180 to RS05205). The *P. agarici* CRISPR/Cas system was identified as comprising of a cluster of six Type I-F CRISPR-associated proteins located downstream of the repeat region. The Cas proteins identified are characteristic of a Type 1 Subtype I-F CRISPR system, similar to that reported in *Pseudomonas aeruginosa* PA14. This involves a CRISPR RNA-guided surveillance complex (also known as a Csy complex), to recruit the Cas2/3 (RS05185) *trans*-acting nuclease for degradation of the target DNA.

In total 34 spacer regions were identified of 32 bp in length. Only one spacer sequence, number 34, showed any nucleotide identity to a known sequence as identified by BLASTn, although a single base substitution was present for the first G, to an A, in the matched sequences. Three matches were found corresponding to plasmid sequences; a plasmid present in *P. frederiksbergensis* strain AS1, one in *P. fluorescens* strain PC20 (plasmid pNAH20) and one in *P. putida* NCIB 9816-4 (plasmid pDTG1) and the single base pair substitution was conserved in all matches. These plasmids were found to encode naphthalene degrading enzymes, including the plasmid originating from *P. frederiksbergensis* strain AS1 which has been demonstrated to be a naphthalene-degrading bacterium. None of the spacer regions showed similarity to the sequences of the phages identified during this study.

In total seven ORFs (RS05570, RS05645, RS18565, RS18570, RS24540, RS18660, and RS18670) were identified that were predicted to encode restriction endonucleases. Of these seven ORFs, three were predicted to encode the restriction (R) subunits (RS05645, RS18570, and RS18670) and two to encode the specificity (S) subunits (RS24540 and RS18670). Two SAM-dependent DNA methyltransferase encoding ORFs were identified that are associated with four of the restriction endonuclease ORFs identified, one (RS18580) which is associated with the restriction endonuclease subunits RS18565 and RS18570, and a second (RS18665) which is located between the S and R restriction endonuclease subunits RS18660 and RS18670.

An operon comprised of 12 genes was identified that is hypothesised to be involved in the production of alginate. All 12 ORFs within this operon are orthologous to the 12 proteins (AlgD, Alg8, Alg44, AlgK, AlgE, AlgG, AlgX, AlgL, AlgI, AlgJ, AlgF, and AlgA) required for alginate production and export in *Pseudomonas* (Table S8).

Pseudomonas sp. NS1

No CRISPR-associated repeats or proteins were identified within the genome of *Pseudomonas* sp. NS1, however four predicted restriction endonuclease proteins (RS15035, RS21265, RS27500, and RS27505) were identified. The protein RS27500 and RS27505 contained McrC and McrB conserved domains, respectively. The McrB conserved domain is associated with the GTP-binding regulatory subunit of the methylcytosine specific McrBC restriction endonuclease of *E. coli* K-12. As the McrBC restriction endonuclease cleaves foreign methylated DNA, it does not possess an associated methyltransferase.

Similar to both *P. tolaasii* NCPPB 2192T and *P. agarici* NCPPB 2472, an operon predicted to encode the genes required for alginate biosynthesis was identified within the genome of *Pseudomonas* sp. NS1 that could possibly act to mask cell surface receptors for phage. In comparison to the operon in *P. agarici*, which contained predicted proteins orthologous to the 12 proteins required for alginate synthesis, the operon in *Pseudomonas* sp. NS1 contained 13 predicted proteins (RS11760 to _11825), and lacked a protein orthologous to AlgE. However, it did contain an additional hypothetical protein in ORF position 12 (Table S8).

### 3.3. Phage Isolation and Characterisation

**Figure 1:**
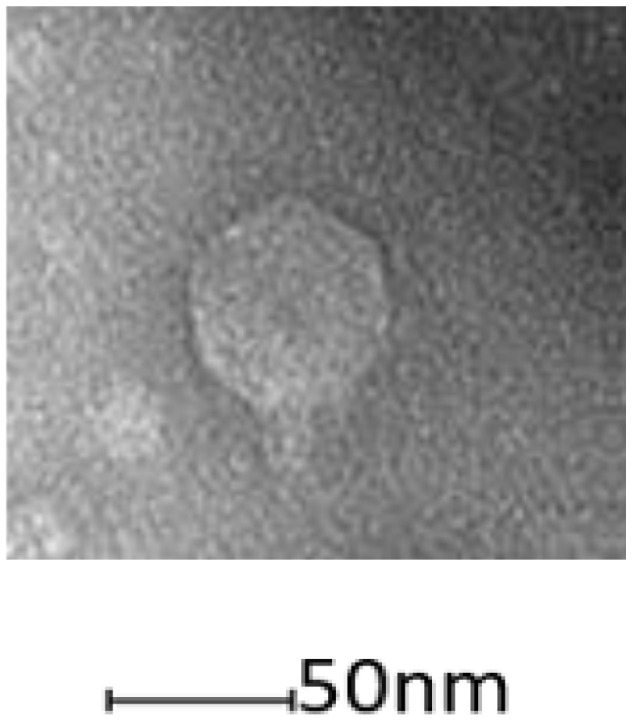
Morphology of phage NV1 by transmission electron microscopy. This phage has podovirus morphology.

To obtain phage that might infect *P. agarici* and *P. tolaasii*, samples were taken from river water and sewage effluent as these are two habitats that regularly contain a large number of diverse phages. Three phages were identified that were able to infect either or both of the mushroom pathogens. All phages exhibited typical head and tail morphologies associated with podoviruses within the order *Caudovirales* (Figure 1). Phage NV1, isolated from a water sample from the River Thames, had a very small plaque size (<2 mm) with a hazy appearance. Phage ϕNV3 isolated from untreated sewage had large clear plaques (5 mm). Phage NV6 was isolated simultaneously with ϕNV3 from the same untreated sewage sample, however the plaques formed on *P. tolaasii* NCPPB 2192T plates were significantly smaller (~1 mm) and cloudy compared to the larger and clear plaques (5 mm) it formed on *P. agarici* NCPPB 2472. All three phages were tested against a range of strains (*P. syringae pv. morsprunorum* R1 5244, *P. syringae pv. syringae* 9097, *P. syringae pv. morsprunorum* R2 5255, *P. fluorescens* PfO-1, *P. aeruginosa* 14,207, and *Pseudomonas* sp. NS1), but showed a remarkably narrow host range, with phages NV1 and ϕNV3, being only capable of infecting *P. tolaasii* NCPPB 2192T and *P. agarici* NCPPB 247, respectively. However, phage NV6, which has morphological similarities to phage ϕNV3, had the ability to lyse both *P. tolaasii* NCPPB 2192T and *P. agarici* NCPPB 2472.

### 3.4. Experimental Coevolution

**Figure 2:**
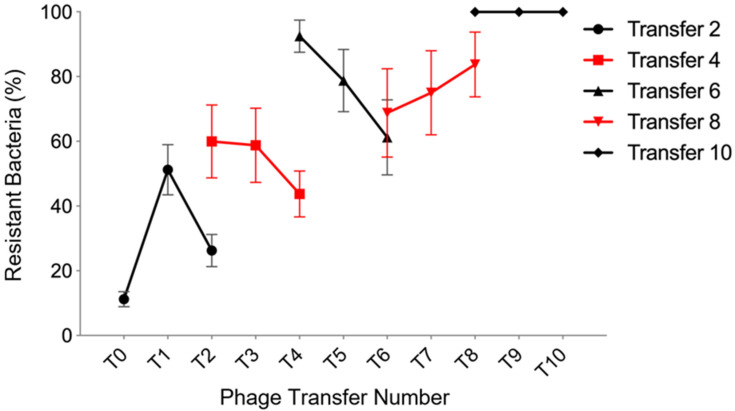
Rate of evolution of phage ϕNV3 through time. The experimental coevolution was performed by inoculating King’s medium B (KB) with phage ϕNV3 and *Pseudomonas agarici* NCPPB 2472. After 48 h incubation at 27 °C, both bacteria and phage were recovered and transferred to fresh KB. This was repeated for 10 transfers. Each line represents the resistance of bacteria to past (the previous transfers), current (the present transfer) and future (the subsequent transfers) phage. Values are mean of 5 replicates +/− SEM.

Understanding coevolutionary interactions between bacteria and phage is an important factor when developing novel phage-based biocontrol applications. To examine the patterns of coevolution between the phages and bacteria, a proportion of the coevolving population was transferred to a fresh microcosm, using sequential transfers of 100 μL of phage NV1 and *P. tolaasii* NCPPB 2192T and phage ϕNV3 and *P. agarici* NCPPB 247 in 6 mL of KB followed by overnight incubation at 27 °C. From this, samples of purified phage and bacteria were taken, before phage infection capacity was measured by streaking on plates of bacterial colonies of each transfer (Figure 2). A high degree of coevolution was evident between the phage and host. For bacterial transfers 1, 3, and 5, it was evident that future phage from the next sequential transfer was more effective in infecting the ancestral host; however, at bacterial transfer 8 the future phage was less effective and by bacterial transfer 9 all bacteria were resistant to all phage tested. Over the course of the experiment escalatory evolution of bacterial resistance was evident in the gradual increase in the percentage of bacteria resistant across all past, present and future phage, resulting in the final total resistance of the bacteria to all phage and eventual phage extinction. The initial bacterial transfer (transfer 1) showed that the future phage (transfer 2) were capable of overcoming the initial bacterial resistance mechanisms, a similar outcome was observed in transfers 4 and 6.

### 3.5. Phage Whole Genome Sequencing

#### 3.5.1. Phage NV1

**Figure 3:**
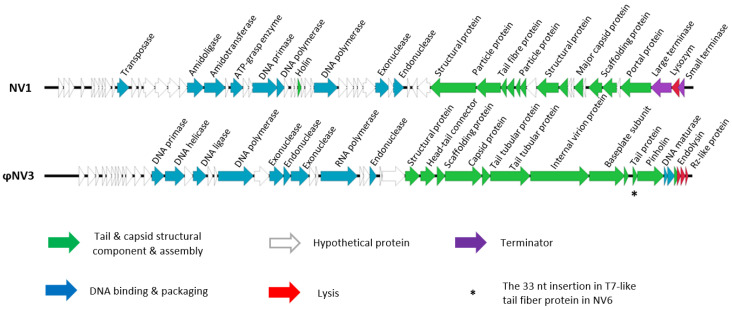
The genome structure of *Pseudomonas* phage NV1 and ϕNV3. Diagram generated using Easyfig [69].

To further analyse the phages and investigate how closely they are related, genomic DNA was extracted, and genome sequencing performed. The NV1 genome was 45,059 bp in length with an average GC content of 52.1%. BLASTn analysis showed NV1 had 84% sequence similarity to the *Pseudomonas fluorescens* phage UFV-P2. The complete genome of phage NV1 was submitted to GenBank with the accession number: MG845684.1 (Figure 3).

Of the 64 predicted ORF’s, only 25 showed amino acid similarity to proteins of known or predicted function as identified by InterPro and BLASTp. The majority of those with predicted functions were those that were involved in virion structure, assembly, and DNA packaging, which showed high levels of amino acid sequence conservation among other closely related phages. The phage NV1 genome possesses a canonical lysis system comprised of a holin and endolysin. In total, 13 potential single-strand nick sequences were identified in NV1 with consensus sequence 5′-TACTRTGAC-3′; which were similar to other *Bruynoghevirus*.

#### 3.5.2. Phage ϕNV3

The ϕNV3 genome was 43,184 bp in length with an average GC content of 58.29%. BLASTn analysis showed ϕNV3 had 77% sequence similarity to phage ϕKMV. Phage ϕNV3 had a T7-like packaging scheme with direct terminal repeats of 693 bp in length identified using PhageTerm [68]. The completed sequence was submitted to GenBank with the accession number MG845683 (Figure 3). The lysis cassette of ϕNV3 is comprised of four proteins; a predicted endolysin, pinholin and Rz-like and Rz1-like proteins as well as the order of these genes indicated that ϕNV3 possesses a SAR-endolysin system conserved in *phikmvviruses*. Analysis of the amino acid terminal sequence of ϕNV3_p44, a putative endolysin, revealed that this region had a large degree of conservation with that of ϕKMV, a member of *Autographiviridae* family, subfamily *Krylovvirinae*. The ϕNV3 N-region of nine amino acid residues, which is three residues longer than that of ϕKMV, has a net positive charge, and thus likely acts as a positive anchor to the negatively charged inner site of the cytoplasmic membrane. The H-region shows a large degree of conservation with that of ϕKMV and is composed of hydrophobic residues, which tend to form an alpha α-helix. This α-helix enhances insertion of the signal peptide into the phospholipid double layer. The catalytic domain shows again a high degree of amino acid sequence conservation between ϕNV3 and ϕKMV with the catalytic residues being identical (Figure S1).

#### 3.5.3. Phage NV6

**Figure 4:**
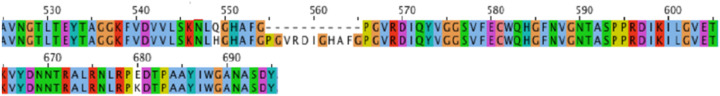
Amino acid sequence comparison of a section of the T7-like tail fiber protein gene of phage ϕNV3 (top) and phage NV6 (below).

The phage NV6 genome was 43,217 bp in length being 99.92% identical to ϕNV3 except for an additional 33 nt when compared to the figure of 43,184 bp for ϕNV3 (Figure 4). Alignment of NV6 sequence to the genome of ϕNV3 by BLASTn indicated that these 33 bases were in a single insertion within a 2367 bp gene predicted to encode a putative T7-like tail fiber protein. The 33 bp addition maintained the single reading frame of the ϕNV3_p40 tail protein and was shown to have added a sequence of 11 aa towards the C-terminal, and an additional two non-synonymous mutations within this gene. Therefore, phage NV6 is a strain of ϕNV3, differentiated by a short insertion in the tail fiber gene.

### 3.6. Lysis Cassette Expression

**Figure 5:**
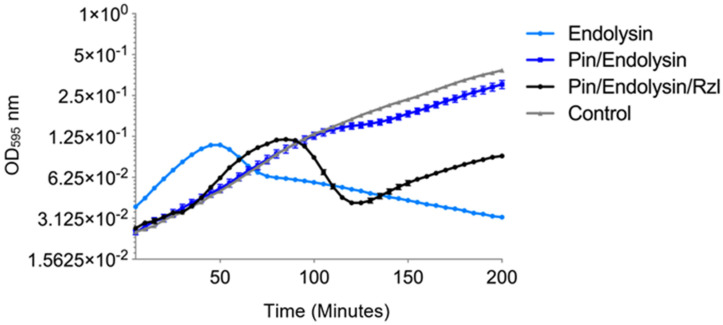
Effect of expression of individual lysis protein constructs containing either the ϕNV3 endolysin, pinholin, or Rz/Rz1-like encoding genes on optical density of BL21-AI *E. coli* following induction with 0.2% L-arabinose at T = 0. The control comprised of an uninduced BL21-AI containing the Rzl/Rz1-like construct. Mean values of 6 replicates are plotted, error bars = +/− SEM.

To gain insight to the process by which these phages lyse bacteria, the lysis cassette was identified and investigated. Bacteriophage lysis cassettes are comprised of the phage encoded proteins involved in lysing the host cell to allow progeny phage escape at the end of the phage replication cycle. The encoded proteins may be comprised of an endolysin and holin, or in the case of SAR-endolysins, the pinholin, lysin and Rzl/Rz1-like proteins. As phage ϕNV3 was predicted to encode a SAR-endolysin system, comprised of four proteins (pinholin, lysin, and Rzl/Rz1-like proteins, Figure S1), it was chosen for the lysis cassette expression study. To confirm the function and lytic activity of the predicted lysis cassette, three genes that encoded pinholin, endolysin, and Rzl/Rz1-like of ϕNV3 were cloned individually in to the pEXP5-CT/TOPO^®^ vector containing a T7 promotor and transformed into BL21-AI™ competent cells, which carries the T7 polymerase gene under control of an arabinose-inducible promotor (*araBAD*) allowing for control over T7 RNA polymerase expression. The effect of expression was tested by inducing expression in exponential phase cells at a starting OD_595_ of 0.4 by adding L-arabinose to a final concentration of 0.2% *w/v* at time zero (T = 0) (Figure 5).

The results of the expression assays showed the strongest decrease in OD, corresponding to a drop in bacterial cell numbers, with expression of the BL21-AI™ *E. coli* containing the endolysin construct. The OD of the BL21-AI™ containing the endolysin construct began to decrease rapidly at T = 50 min, with a 43% reduction at T = 80 min; the OD continued to gradually decrease over the remaining 120 min at a reduced rate, a total decrease of approximately 70%.

For the pinholin expression assay, the OD continued to rise at a similar rate to the control until 80 min. The OD began to rise again after 115 min, however at a lower rate. The final OD at 200 min was 0.28, significantly higher (*p* < 0.001) than the endolysin expression (OD of 0.032) (Figure 5).

**Figure 6:**
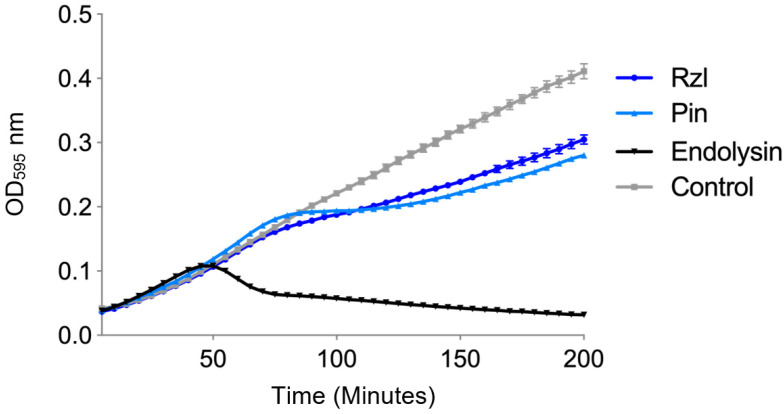
Complementation assay of lysis cassette proteins of ϕNV3 by expression of constructs containing either the ϕNV3 endolysin gene, pinholin and endolysin genes or the complete lysis cassette in BL21 AI *E. coli* following induction with 0.2% L-arabinose at T = 0. The control comprised of an uninduced BL21-AI containing the Pin+Endolysin construct. Mean values of 6 replicates are plotted, error bars = +/− SEM.

For the Rz/Rzl-like expression, the OD was similar to the control until 75 min post induction, where it began to rise at a reduced rate in comparison to the control. However, the OD was higher than the OD of the strain expressing the pinholin (Figure 5). In order to determine the effects of pinholin and Rz/Rz1-like protein on the ability of the endolysin protein to lyse the host cells, a further two constructs were created containing either the pinholin and endolysin or the full lysis cassette (pinholin, endolysin and Rzl/Rzl-like protein) and expressed in BL21-AI™. The expression of the endolysin alone showed the most rapid increase in OD across all induced constructs (Figure 6). The increase in OD continued to a peak at T = 50 min followed by a rapid drop, indicative of cell lysis, with a 41% decrease by the end of the experiment. In contrast, the OD, in the complete lysis cassette construct expression, was similar to the control until T = 40 min. The OD then increased rapidly to a peak at T = 85 min, with a rapid decrease of ~66% at T = 120 min. The OD began to rise slowly over the remaining time of the experiment. The OD of the bacterial culture expressing both the pinholin and endolysin increased at the same rate of the control up until T = 105 min, but increased at a slower rate when compared to the control. Taken together, these indicate that the ϕNV3 endolysin protein is capable of causing cell lysis in the absence of the pinholin and Rz/Rzl-like proteins, although the greatest drop in bacterial cell numbers is correlated with the expression of full lysis cassette protein.

## 4. Discussion

The *Pseudomonas* genus is large and complex, including a variety of species that live in a wide variety of habitats and can have neutral, beneficial, or detrimental effects on other organisms. Several *Pseudomonas* species cause disease in mushrooms, including *P. tolaasii* and *P. agarici* that cause different pathologies. We aimed to examine the genome sequences of *P. tolaasii* and *P. agarici* to look for clues on how they might cause disease as well as isolating and characterising phages that target these strains. During this study we also isolated a non-pathogenic *Pseudomonas* strain from the surface of *A. bisporus* mushroom showing no symptoms of disease. Therefore, we used it as a comparator for the other two pathogenic strains in both genome comparison and for phage testing.

The genome of *P. tolaasii* NCPPB 2192T, *P. agarici* NCPPB 2472, and *Pseudomonas* sp. NS1 were analysed for potential biosynthetic gene clusters involved in the production of virulence factors, including toxins such as tolaasin [70], or siderophores, such as pyoverdines [24]. Within the genome of *P. tolaasii* NCPPB 2192T, 6 ORFs were identified with significant amino acid identity to the proteins TaaA-TaaE of *P. costantinii,* which have been demonstrated by Scherlach, et al. [32] to be involved in the production of Tolaasin I. Two of the further NRPSs identified within the genome were found to be likely consist of a single large NRPS with amino acid identity, to the known pyoverdine biosynthesis genes PvdJ of *P. syringae pv. daphniphylli* and the pyoverdine sidechain peptide synthetase of *P. fluorescens*. Further, an ORF downstream of these NRPSs was also found to show amino acid identity to the PvdJ protein of *P. aeruginosa* strain 62. Pyoverdines are fluorescent siderophores that are important virulence factors in many fluorescent *Pseudomonas* [24,71]. The presence of a TonB-dependent siderophore upstream of these putative NRPSs and their similarity to known pyoverdine synthesis proteins indicates this cluster is likely to be involved in the synthesis of pyoverdine or related chromophore.

A large biosynthetic cluster was identified within the genome of *P. agarici* NCPPB 2472, which was predicted to encode the proteins involved in the synthesis of the siderophore achromobactin. Achromobactin production was first identified by Munzinger, et al. [72] in the bacterium formally known as *Erwinia chrysanthemi* (now *Dickeya dadantii* [73]), as a siderophore derived from the precursor citrate. Berti and Thomas [74] reported that *P. syringae pv. syringae* B728a was capable of achromobactin synthesis, which was produced via NRPS-independent synthetases; demonstrating that the enzymes AcsD, AcsA, and AcsC were capable of converting citrate to achromobactin [74]. We identified a biosynthetic gene cluster in *P. agarici* NCPPR 2472, which was predicted to encode the enzymes required for achromobactin synthesis, which showed similarity in ORF organisation to the cluster of *P. syringae pv. syringae* B728a, as well as a high degree of amino acid identity (72–91%) between the predicted gene products. We therefore hypothesised that *P. agarici* NCPPB was likely to produce the siderophore achromobactin under iron-limited conditions. Both *D. dadantii* and *P. syringae pv. syringae* B728a produce a second siderophore in addition to achromobactin [72,74,75], with the primary siderophore, achromobactin, required for epiphytical growth and that the secondary siderophores reserved for active infection [74,76,77]. It was therefore possible that one of the identified NRPS clusters of *P. agarici* NCPPB 2472 was involved in production of a siderophore, such as pyoverdine, utilised in active infection of *A. bisporus,* and that the predicted achromobactin siderophore was utilised during non-infective growth in the environment.

Within the genome of *Pseudomonas* sp. NS1, 10 complete or partial NRPSs were identified, 8 of which resided in two distinct clusters. A single cluster containing 6 NRPSs showed nucleotide identity to the WLIP production cluster 2 of *P. fluorescens* strain LMG 5329. WLIP, a subtype of the viscosin-related nonapeptides [78], produces a precipitate in agar medium when a WLIP producing *Pseudomonas* strain was co-cultured alongside a strain of tolaasin producing *P. tolaasii* [78]. A second, ORFan, NRPS showed nucleotide identity to WLIP production cluster 1 of *P. fluorescens* LMG 5329, which has been identified by Rokni-Zadeh, et al. [79] to be conserved in both the WLIP NRPS system and the viscosin system of *P. fluorescens* SBW25. Further analysis revealed high similarity between the NRPSs within the *Pseudomonas* sp. NS1 cluster and the WLIP synthetases of *P. fluorescens* LMG 5329 led to the hypothesis that the identified gene cluster was likely to encode the proteins required for WLIP synthesis. This hypothesis was tested by co-culturing *Pseudomonas* sp. NS1 alongside *P. tolaasii* NCPPB 2192T, revealing a white precipitate, confirming that *Pseudomonas* sp. NS1 was capable of WLIP production and that was most likely produced by the NRPSs and associated proteins identified.

Although a wide variety of secondary metabolites involved in host colonisation and invasion by *Pseudomonas* species are produced by NRPS’s, many are produced by more traditional assembly routes; therefore, further biosynthetic clusters that may confer advantages on the host surface were identified and characterised, including putative phage tail-like bacteriocin (PTLB) encoding genes. PTLBs are bacteriocins of which there are three broad types, F, R, and S [80]. Both the F and R type PTLBs resemble bacteriophage tail proteins, with the R type being most similar to contractile phage tails [81,82] and the F type being flexible non-contractile rods similar to flexible phage tails [82] and are likely both of phage origin [83]. PTLBs are very common in *Pseudomonas* strains, with 90% *P. aeruginosa* strains being reported to produce either the F or R type [84]. Putative PTLB encoding operons were identified in both *P. tolaasii* 2192T and *P. agarici* NCPPB 2472, with the operon for *P. agarici* NCPPB 2472 consisting of a total of 19 ORFs and the smaller operon for *P. tolaasii* NCPPB 2192T consisting of only 13 ORFs in comparison.

To determine if it was possible to identify potential bacterial gene systems that promote colonisation and pathogenicity of mushrooms, we searched for the typical protein secretion systems that can underpin a pathogenic lifestyle on eukaryote hosts: these include the type III and type IV protein secretion systems that can inject effectors into host cells and type VI secretion system that can be used for competition with other bacteria whilst also potentially impacting the host [14]. Type III protein secretion systems underlie the pathogenicity of many Gram-negative bacteria, such as plant pathogenic *Pseudomonas*. The *hrp*-*hrc*-encoded type III secretion system, which injects bacterial effector proteins (historically called Hop or Avr proteins) into plant cells, is required for pathogenicity [85]. Effectors are also host range determinants whose presence can be specifically recognized by plants [86]. We found both mushroom pathogens and the non-pathogenic strain NS1 had between one to three T3SS, with elements of broken up T3SS clusters also being evident in the genomes. Looking at the gene synteny, the T3SS in the three genomes had similarity to the T3SS of *P. fluorescens* with the presence of *rspL* and *rspR* but no *hrpS* homolog as found in *P*. *syringae*. A *hrpZ1* harpin gene was observed in one of the *P*. *agarici* clusters, potentially indicating involvement in membrane-binding and pore-forming activities as seen with *P*. *syringae* [87]. The BLAST analyses indicated the genes were most closely related to strains in the *P*. *fluorescens* species complex including the ginger blotch mushroom pathogen, *P*. *gingeri* [9]. Very few effector genes were found in any of the strains with *hopJ* being present in all three strains, *hrpK* being present in the two pathogens (two copies in *P*. *agarici*), *exoU* being present only in *P*. *tolaasii* and strain NS1 while *hopAC1* was present only in *P*. *agarici*. Given the differences in pathology, it would certainly be interesting to determine whether any host specificity or niche specificity is governed by these differences, though it would also be important to keep in mind that these clusters may play roles in other eukaryote interactions [88,89]. A particularly intriguing observation was the presence in *P*. *agarici* of a third, quite different T3SS (RS22985–23120) resembling the Inv-Spa gene cluster found in human pathogens [90,91]. This would also be a good target to examine whether the cluster plays a role in the drippy gill disease or is involved in its wider ecology such as interactions with alternative hosts [92].

Type IV pili are surface filamentous appendages expressed by many pathogenic bacteria [93], which play a role in bacterial adherence to and invasion of various types of host cells [94]. *pilW*, *pilI,* and pilin genes were identified in *P. tolaasii* NCPPB 2192T, *pilW*, *pilX, pilG*, *pilT*, *pilB,* and pilus assembly protein genes were found in *P. agarici* NCPPB 2472, and *pilQ* and *pilV* were found in *Pseudomonas* sp. NS1. These observations suggest none of the strains encode a full complement of genes for making a pilus, but this will need to be examined further.

T6SS have been implicated in the pathogenicity of several Gram-negative bacteria as well as being important for interbacterial competition [95,96]. Large type VI gene clusters were found in all three strains. Hcp (hemolysin-coregulated protein) and VgrG (valine glycine repeat) represent key components or effector proteins of type VI [97]. Hcp1 was identified in all three genomes, five in *P. tolaasii* NCPPB 2192T, and six in *P. agarici* NCPPB 2472. Three VgrG genes were found in *P. tolaasii* NCPPB 2192T and two Hcp1 and VgrG were identified in *Pseudomonas* sp. NS1 genome. Together, these observations highlight differences in the three strains that can be examined for contributions to colonisation and disease.

The genome of *P. tolaasii* NCPPB 2192T, *P. agarici* NCPPB 2472 and *Pseudomonas* sp. NS1 were analysed for the presence of prophage. The bacterial host infection by a temperate phage can lead to lysis or lysogeny. During lysogeny, temperate phage either integrate their genome as a “prophage” into the host genome where it is replicated by the host during cell division or form autonomous plasmids within the host [80]. The prophage may carry and express genes that benefit and increase fitness of the host cell by a process known as lysogenic conversion [98]. It has been shown that prophages can encode genes that confer phage resistance through a variety of mechanisms [99]. Many prophages encode superinfection exclusion proteins that inhibit further phage infection by altering the bacterial cell envelope, which have been characterised in *Escherichia coli* phages HK97 [100], φ80 [101], *Salmonella* phage P22 [102], and *Streptococcus* phage TP-J34 [103]. The genome of *P. tolaasii* NCPPB 2192T contained five active prophages with similarity to other phages including *Pseudomonas* phage UFV-P2 (NC_018850.2) and it would be useful to assess their role in contributing to phage resistance, pathogenicity, and environmental persistence. *Pseudomonas* sp. NS1 genome had one prophage with similarity to *Escherichia* phage vB_EcoM_ECO1230-10 (NC_027995.1). However, these predictions require experimental confirmation to fully confirm their presence in the genome.

The genomes of *P. tolaasii* NCPPB 2192T, *P. agarici* NCPPB 2472 and *Pseudomonas* sp. NS1 were also analysed for the presence of phage resistance genes that may prevent or hinder the possible use of bacteriophage in the treatment of *A. bisporus* mushrooms. Within the genome of *P. agarici* NCPPB 2472, we identified multiple phage resistance systems, including a complete CRISPR/Cas system. No confirmed CRISPRs or CRISPR-associated proteins were identified in the genomes of the closely related *P. agarici* NCPPB 2289, *P. agarici* LMG 2112, or within either of the genome sequences of *P. tolaasii* NCPPB 2192T or *P.* sp. NS1. The Cas proteins of *P. agarici* NCPPB 2472 were characteristic of a Type 1 Subtype I-F CRISPR system which have also been documented in *P. aeruginosa* strain PA14 [104], and strain UCBPP-PA14 [105], *Pectobacterium atrosepticum* [106] as well as certain strains of *E. coli*, where it was reported to be more frequently found in strains susceptible to antimicrobials, possibly due to interference in resistance plasmid acquisition [107]. The repeat consensus identified in *P. agarici* NCPPB 2472 was 28bp in length and a total of 33 spacer regions were associated with the identified repeats. Of the 34 spacer sequences, only a single sequence showed any similarity to known sequence, with a single base pair substitution. This sequence corresponded to three plasmid sequences present in the *Pseudomonas* strains: *P. frederiksbergensis* strain AS1, *P. fluorescens* strain PC20 (plasmid pNAH20), and *P. putida* NCIB 9816-4 (plasmid pDTG1). The identified plasmid sequences were found to encode naphthalene degrading enzymes, including the plasmid that was identified from *P. frederiksbergensis* strain AS1, which been demonstrated to be a naphthalene-degrading bacterium [108]. None of the spacer regions showed any nucleotide identity to the genomic sequences of any of the phage used in the course of this study.

While only *P. agarici* NCPPB 2472 was identified as possessing a CRISPR/Cas system, all of the *Pseudomonas* strains sequenced in this study possessed restriction endonuclease systems, which are a well-documented method by which bacteria are able to resist phage infection [109]. Within the genome of *P. tolaasii* NCPPB 2192T several resistance endonucleases were identified. The restriction endonucleases identified included a probable methylation-dependent restriction endonuclease, RS17360, and a Type III restriction endonuclease, RS17410. This shows that *P. tolaasii* NCPPB 2192T possesses defence mechanisms against phage infection, including by phages with previously methylated nucleotides. Similarly, within the genome of *Pseudomonas* sp. NS1, two restriction endonuclease proteins (RS27500 and RS27505), were identified as containing McrC and McrB conserved domains respectively, which are associated with the methylcytosine specific McrBC restriction endonuclease of *E. coli* K-12 [110]. This indicates that similar to *P. tolaasii* NCPPB 2192T, *Pseudomonas* sp. NS1 is capable of cleaving previously methylated nucleotides. In comparison while multiple restriction endonucleases were identified within the genome of *P. agarici* NCPPB 2472, including two Type I restriction endonuclease R subunits (RS18570 and RS18670), no methylation-dependent restriction endonucleases were identified.

A second potential phage resistance system was identified in all three *Pseudomonas* species in this study. While not a specific phage resistance system, alginate can act to mask host receptors from phage, and therefore can offer an advantage to the producing bacterium [111]. For *P. tolaasii* NCPPB 2192T a complete operon of 15 genes, was identified that was predicted to be involved in the production of alginate due to the presence of a gene encoding the alginate biosynthesis protein Alg44, which has been demonstrated to be required for alginate biosynthesis in *P. aeruginosa* [112] and AlgE, also reported to be involved alginate biosynthesis in *Pseudomonas* [113]. Within the genome of *P. agarici* NCPPB NCPPB 2472, we identified a 12 ORF operon which was comprised of proteins orthologous to the 12 proteins required for alginate production and export in *Pseudomonas* [112]. Therefore, it is highly likely that *P. agarici* NCPPB 2472 was capable of producing alginate. Similar to the operon of *P. agarici* NCPPB 2472, a cluster of 13 genes was identified in the genome of *Pseudomonas* sp. NS1, which are required for alginate production and export in *Pseudomonas* [113]; although, unlike the operon identified in *P. agarici* NCPPB 2472, it lacked a protein orthologous to AlgE and also contained an additional hypothetical protein in ORF position 12. However, the presence of 11 out of the 12 genes required for alginate production and export indicated this cluster was highly likely to be involved in the production of alginate in *P.* sp. NS1.

Another aim of this study was to isolate phages that could infect the mushroom pathogenic *Pseudomonas* species to explore their potential application in biocontrol. On lawns of *P. tolaasii* NCPPB 2192T, phage NV1 formed very small hazy plaques, historically associated with either a temperate (lysogenic) life cycle, chronic infection [114], or inefficient phage adsorption to the host [115], but no genes associated with lysogeny were identified within the NV1 genome. However, Roach, et al. [116] demonstrated that production of hazy plaques, by phages infecting plant pathogenic *Erwinia amylovora*, is an indication of preference of phages to low and high exopolysaccharide producing host cells. For example, *Myoviridae* phages produced clear plaques on low-exopolysaccharide-producing hosts and turbid plaques on high-exopolysaccharide-producing hosts. The reverse preference was seen by most *Podoviridae* phages, where clear plaques were seen on high-exopolysaccharide-producing hosts.

The genome of NV1 revealed 84% nucleotide identity with the *P. fluorescens* phage UFV-P2. The International Committee on Taxonomy of Viruses (ICTV) guidelines recommend DNA sequence identity of 95% as a threshold for species delineation, with phage NV1 showing a maximum nucleotide identity below the species delineation threshold [117]. The differing host specificity, GC content and genome size would indicate that phage NV1 is a new species. ICTV taxonomy has grouped both phage NV1 and UFV-P2 into the genus *Vicosavirus* in *Podoviridae* family (https://talk.ictvonline.org/taxonomy/).

The genome of ϕNV3 showed 75% similarities with *Pseudomonas* phage ϕKMV [118]. Similar to phage NV1, the majority of genes with identified functions were phage structural proteins and those involved in DNA replication, such as the single subunit RNA polymerase upstream of DNA replication genes, adjacent to the structural protein region of the genome, which was characteristic of the *Autographiviridae* family and similar to phage ϕKMV and other ϕKMV-like phages [81,119]. ICTV taxonomy has placed Phage ϕNV3 in *Kirikabuvirus* genus, *Autographiviridae* family. However, phage ϕKMV is placed in *Phikmvvirus*, *Autographiviridae* family.

Phage NV6, was initially identified on mixed culture plates of *P. tolaasii* NCPPB 2192T from untreated sewage and was originally thought to be a unique phage; it was able to cause plaques on both *P. tolaasii* NCPPB 2192T and *P. agarici* NCPPB 2472, with morphological and sequence similarities to phage ϕNV3. Genome analysis identified a single addition of 33 bases in NV6 genome compared with ϕNV3, within an ORF encoding a putative T7-like tail protein. It was therefore likely that this region within the T7-like tail fiber protein is potentially involved in receptor binding and a determinant of host specificity, as demonstrated by the broader host range of NV6 compared to ϕNV3. Boon, et al. [119] showed that the host range expansion of *Pseudomonas* phage LUZ7 was due to a conserved tail fiber mutation. This mutation allowed the phage to adsorb to *P. aeruginosa* strains that were not originally recognized by the wild-type phage.

The coevolutionary experiment, to coevolve phage and bacteria together to check if new phage genotype would emerge to overcome bacterial resistance and vice versa, showed that up to 51% of bacteria developed resistance to the current phage transfer within 24 h cycle. However, they appeared to be less resistant to future phage for bacterial transfer numbers 1, 3, and 5, which may indicate that the development of phage resistance by *P. agarici* NCPPB 2472 at this stage comes at cost [19]. This pattern can indicate an arms race under fluctuating selection dynamics. This pattern can be seen in the gene-for-gene pattern of coevolution [120,121]. The resistance to present phage increased to 79% by transfer 5 and by transfer 9 the bacteria were resistant to all past, present, and future phage; this complete resistance to all phage assayed in transfer 9 might be associated with the acquisition of a spacer region associated with phage ϕNV3 in the CRISPR/Cas system of *P. agarici* NCPPB 2472. Another explanation might be the presence of a mutator strain of the bacterium within a population which is likely to cause the extinction of the phage population. Whilst these data suggests ϕNV3 may not be suitable for use as a biocontrol agent of *A. bisporus* blotch disease in pure preparation, it may be possible to avoid the rapid evolution of resistance if it is included as part of a phage cocktail [122]. However, the frequency of bacteriophage-insensitive mutants should be assessed to fully understand the coevolutionary results.

While phage treatments of bacterial infections have been well documented in literature [123], phage lysis proteins have also been studied intensively as potential enzybiotics in the treatment of bacterial infections [123,124,125] and have shown promise in vivo [126]. Furthermore, artilysins, modified phage endolysins with specific outer membrane-destabilizing peptides, are being used for exogenous application of endolysins to control bacterial pathogens in situ [127]. For this reason, the lysis cassette of ϕNV3 was characterised. Phage ϕNV3 possesses a SAR-endolysin system comprised of four proteins, the pinholin, endolysin, Rz and Rzl-like proteins, a system that is conserved in all *phikmwviruses* [128] and differing from the canonical system putatively identified in phage NV1. The SAR-endolysin system of ϕNV3 were almost identical with that of ϕKMV, especially within the N-terminal “signal-arrest-release” domain of the endolysin protein as well as sharing conserved catalytic residues within the catalytic domain. These catalytic residues (E, D, and T) are of the conserved protein domain family of the endolysin R21-like proteins (cd16900) which are muralytic enzymes and cleave the glycosidic beta 1,4-bonds between the N-acetylmuramic acid and the N-acetylglucosamine of the peptidoglycan [129]. This is further confirmed by the similarity to endolysin KMV45 which also possesses muralytic activity [128]. This also highlights the N-terminal signal domain. This signal peptide sequence is what allows the secretion of the endolysin with the host Sec secretory system, that allows access to the peptidoglycan, independent of a classical holin [128]. Briers, et al. [128] showed that the endolysin KMV45 protein alone when expressed was able to cause cell lysis after 60–90 min, which correlates well with the observed 43% reduction at 80 min seen with ϕNV3 constructed in this study. This would indicate that the lysis cassette of ϕNV3 likely operates with a similar mechanism as that reported for ϕKMV by Briers, et al. [128]. The endolysin of *Thermus scotoductus* phage vB_Tsc2631 contained a positively charged N-terminal extension of short residues that protruded from the remainder of the enzyme and was crucial for peptidoglycan binding [130]. Moreover, antibacterial activity of lytic enzyme LysC, from *Clostridium intestinale* URNW, was mediated by its highly positively charged N-terminal region [131].

In order to confirm the predicted function of the endolysin proteins and associated proteins of the lysis cassette, the proteins were cloned into the pEXP5-CT/TOPO^®^ vector and transformed in to BL21-AI™ cells for expression. The results confirmed the lytic ability of the ϕNV3 endolysin protein, even without the presence of the pinholin and Rz/Rzl-like proteins, although the greatest drop in bacterial cell numbers was correlated with the full lysis cassette protein expression although the time required for this drop was longer in comparison to the endolysin protein expressed alone; however, this may be attributed to the metabolic pressure of expressing multiple proteins simultaneously compared to a single endolysin protein. Expression of the lysis proteins of NV1 were also performed which demonstrated limited differences in optical density between the induced and uninduced controls, which might be due to the different lysis systems involved.

## 5. Conclusions

In this study we isolated novel phages that are able to lyse the pathogenic bacteria *P. tolaasii* and *P. agarici*, but not the non-pathogenic mushroom resident *Pseudomonas* sp. NS1, and thus have the potential to be used as biological control of blotch diseases of mushroom. We discovered that phage NV6 had a broader host range probably due to the 33 bp addition in its T7-like tail fiber protein. Clearly, the phages demonstrate an adaptation that affects their host range, presumably due to selection to overcome bacterial resistance, and thus this could be problematic for use as a biocontrol agent. It would therefore be important to work on the development of a phage cocktail to inhibit the emergence of phage-resistant bacteria and to broaden the utility for phage formulations to treat specific bacterial pathogens of mushroom. Furthermore, our work showed that the lysis cassette of one of these phages is effective at lysing bacteria and could be exploited for use in control of bacterial populations [132]. We also compared the newly isolated *Pseudomonas* sp. NS1 to the mushroom pathogens. The non-pathogenic *Pseudomonas* sp. NS1 genome exhibited subtle differences compared to the pathogens in relation to secondary metabolite production for toxins, siderophore cluster and phage tail-like bacteriocin. This strain should prove useful in helping to unravel the mechanisms that differentiate mushroom colonisation from pathogenesis. The *Pseudomonas* sp. NS1 was also not affected by any of the phages isolated in this study, providing an indication that these phages do not harm non-pathogenic mushroom residents, but clearly further testing on a larger number of strains will be needed to assess this properly. It would however be interesting to examine the competitive fitness of *Pseudomonas* sp. NS1 against *P. tolaasii* and *P. agarici* to determine whether a joint use of bacteria and phage could be used to restrict pathogen proliferation on the mushrooms.

## Figures and Tables

**Table 1:** Whole genome sequence characteristics of bacteria used in this study.

Strain	GenBank Accession Number	Genome Length (bp)	ORFs	Encoding	RNA	tRNA	rRNA	ncRNA	GC (%)
*P. tolaasii* NCPPB 2192T	CP020369.1	6,856,683	6286	6065	63	56	3	4	60.5
*P. agarici* NCPPB 2472	CP014135.1	5,502,003	4901	4673	67	59	4	4	58.89
*Pseudomonas* sp. NS1	CP022960	6,702,516	6241	6073	72	60	8	4	61.08

## Supplementary Materials

The following are available online at https://www.mdpi.com/1999-4915/12/11/1286/s1. Results: Type III, Type IV and Type VI secretion systems, Table S1: Primers used in NV1 and ϕNV3 lysis protein constructs in this study, Table S2: Table of all lysis protein constructs used in this study, Table S3: Plasmids used in this study, Table S4: Predicted secondary metabolites and non-ribosomal peptide synthetase in *Pseudomonas tolaasii* 2192T, *P. agarici* NCPPB 2472 and *Pseudomonas* sp. NS1 genome, Table S5: Predicted Type III (T3SS), IV (T4SS) and VI (T6SS) secretion systems in *Pseudomonas tolaasii* 2192T, *P. agarici* NCPPB 2472 and *Pseudomonas* sp. NS1 genome, Table S6: Predicted prophage in *Pseudomonas tolaasii* 2192T, *P. agarici* NCPPB 2472 and *Pseudomonas* sp. NS1 genome, Table S7: Predicted phage tail-like bacteriocin in *Pseudomonas tolaasii* 2192T, *P. agarici* NCPPB 2472 genome, Table S8: Predicted phage resistance systems in *Pseudomonas tolaasii* 2192T, *P. agarici* NCPPB 2472 and *Pseudomonas* sp. NS1 genome, Figure S1: lysis cassette of phage ϕNV3.
