# Supplementary material for: Genomic Characterisation of Mushroom Pathogenic Pseudomonads and Their Interaction with Bacteriophages
Source: Viruses. 2020 Nov 10;12(11):1286. doi: 10.3390/v12111286 (PMC7696170; doi:10.3390/v12111286)
Supplement: Supplementary file 1 [file viruses-12-01286-s001.pdf]

viruses MDPI

Supplementary Material Results and Tables

Results

Type III, Type IV and Type VI secretion systems

P. tolaasii NCPPB 2192T

BLASTp analysis of the different secretion systems indicated *P. fluorescens* as the closest hits. Screening of the genome sequence with KofamKOALA and Artemis identified one putative T3SS cluster of 23 genes, from RS00335 to RS00445, and three non-contiguous parts of a T3SS: RS04585 (*hrcC*) as part of a potential operon to RS04600; RS05825 to RS05850 (including a *hrcV* gene); RS12630 to RS12695 (with genes including *hrcC* and *hrcT* homologs).

The complete cluster was located between a putative tRNA-Gln (predicted by tRNAScan-SE) at RS00310 and a putative tRNA-Met (RS00450) with genes for hypothetical protein (HP)-isomerase-*ggt* gene downstream. The genes appeared to be organised into three or four operons and drops in the %GC ratio were observed upstream of RS00335 and RS00440. A putative sigma-54-dependent enhancer binding protein transcriptional regulator (RS00335) with a 75% match (amino acids) to RspR was on the left flank of the cluster and a putative extracytoplasmic function alternative sigma factor RspL (related to HrpL from *P. syringae*) gene (RS00445) with 91% match was found on the right flank. A potential RspL (HrpL) promoter (consensus sequence: GGAACC-15/16N-CCAC) was identified upstream of RS00440 (HP) linked to *hrcV*. Together, these features suggest the cluster is most similar to the gene cluster type found in *P. fluorescens* [1, 2].

The three fragmented genomic regions lacked obvious RspL or RspR genes or RspL (HrpL) promoters, though two potential degenerate promoters were found upstream of two gene clusters, at RS05825 and RS12655. The putative HrcC encoding gene (RS04585) was contiguous with three downstream genes, a HP, a putative Invasion protein (IagB) and quinone-dependent dihydroorotate dehydrogenase. Together, these observations may, therefore, indicate that either the second T3SS cluster is in degradation or that it is adapting for other purposes. A search for T3SS effectors found, like observed for *P. fluorescens*, little evidence of a large complement of genes with only HopJ (RS05495), HrpK (RS09075) and ExoU (RS13030) matches.

A search for a T4SS found a small gene cluster between RS08725-08745 and several other genes spread throughout the genome: *pilW* (RS10610), pilin (RS27580 and RS27585) and *cpaB* (RS27590). Searches for a T6SS identified two operons, in opposing directions, spanning RS14450-14570 and five potential *hcp1* genes (RS26990, RS16835, RS25755, RS14530 and RS18935). Several VgrG alleles (RS00795, RS04750 and RS15065) were also found. Other potential genes were found between RS05335-5375, RS25095-25170, and RS08870 (*vask*) and RS25775 (Supplementary Material Table 5).

P. agarici NCPPB 2472

BLASTp analysis of the different secretion systems indicated the most similarity with *P. gingeri* and in some cases *P. fluorescens* or *P. syringae*. Screening of the genome sequence identified three T3SS gene clusters, from RS06355-006465, RS18850-18985, and RS22985-23120. Large drops in the %GC ratio were observed flanking three regions, but no tRNA genes were observed on either side of them. The RS06355-006465 cluster consisted of 22 genes with genes exhibiting similarity to *P. fluorescens* as well as *P. syringae*, but there appeared to be a loss of synteny with these gene clusters in some sections. The RS18850-18985 cluster consisted of 28 genes with similarity to *P. gingeri* genes. Notably this gene cluster encodes a putative harpin gene, *hrpZ1* as well as a putative *hrpL* (RS18855). The third gene cluster (RS22985-23120) consisted of 27 genes and showed high similarity to a gene cluster in *P. gingeri* annotated as an Inv-Spa cluster of genes. Notably, putative invasion proteins were also observed in this cluster (RS23015-23020 and RS23115-23120), with RS23045 encoding a putative IagB similar to that seen in *P. tolaasii*. These genes are also linked to a putative quinone-dependent dihydroorotate

dehydrogenase gene (RS23005) on one flank thus showing a similar feature as the quinone-IagB linkage in *P. tolaasii*. A gene for a putative SipB effector was also found to be part of this gene cluster. Only three other potential effector genes were identified in the genome: HopJ (RS03325), HrpK (RS18850) and HopAC1 (RS07770), with similarity to *P. gingeri* (all >90% identity). Neither cluster 1 or 3 had obvious T3SS transcriptional regulators associated with them nor were potential HrpL promoter sequences identified in the genome except for *hrpK* and *hopAC1*.

A search for a T4SS found a small gene cluster between RS22660-22685 (including *pilW*, *pilX* and pilus assembly protein genes) and several other genes spread throughout the genome: *pilG* (RS01585), *pilT* (RS01635, RS06650), *pilB* (RS16265) and *pilW* (RS17195).

Searches for a T6SS identified three large gene clusters: RS02645-02770 was arranged in two operons, in opposing directions, with the RS02710-02770 cluster being flanked by large %GC drops; RS21080-21155 was arranged in two operons, in opposing directions, with an Rhs gene on one side (RS21150); RS21510-21580 was arranged as one operon and next to an 8kb region (RS21475-21505) containing a transposase, resolvase and integrase. The entire region is flanked by low %GC ratio drops. A smaller cluster was found at RS12550-12585 and flanked by low %GC ratio regions. A potential type VI-secreted lysozyme-like gene (RS09100) was found. Five genes annotated as Hcp1 Type VI effectors (RS01160, RS02725, RS09880, RS19150, RS19520) were identified as well as four Rhs genes (RS01355, RS05175, RS13620, RS20175) and several pseudogenes (Supplementary Material Table 5).

Pseudomonas sp. NS1

BLASTp analysis of the different secretion systems indicated *P. fluorescens* as the closest hits. Screening of the genome sequence identified two T3SS gene clusters, from RS10335-10445 (23 genes) and RS25340-25440 (21 genes). Large drops in the %GC ratio were observed flanking both regions. A putative tRNA-Met (RS10330) was observed on the left flank of the first gene cluster. Notably, two genes (RS10325-10320) were observed on the other side of the tRNA-Met, a putative phenazine biosynthesis protein, PhzF, and *ggt*. Together there is some syntenic similarity to *P. tolaasii*. The T3SS genes appeared to be organised into two to four operons with gene matches most closely matching *P. fluorescens* F113. A putative RspL (HrpL) gene (RS10335) with 97% match was found on the left flank of the cluster next to the tRNA-Met and a putative sigma-54 transcriptional regulator (RS10445) with a 77% match (amino acids) to RspR was on the right flank. A potential RspL (HrpL) promoter was found upstream of RS10340 (*hrpJ*) and RS10440.

The RS25340-25440 cluster consisted of 21 genes with genes with two potential operons one strand separated by a tetratricopeptide repeat protein gene (RS25410) on the opposing strand. No obvious transcriptional regulators or RspL promoters were observed. On the right flank, after the low %GC region, were three genes encoding putative *luxR* regulator, superoxide dismutase and peroxidase, showing a synteny similar to *P. tolaasii*.

Also similar to *P. tolaasii* was the discovery of an orphan *hrcC* gene (RS29245) contiguous with downstream genes (four instead of three), a HP, transcriptional regulator, *iagB* and quinone-dependent dihydroorotate dehydrogenase genes.

Only two potential T3SS were found, HopJ (RS00750) and ExoU (RS04255), both of which had similarity to *P. fluorescens* and *P. costantinii* (>90%).

A search for a T4SS found a small gene cluster between RS08735-08750 (including *pilQ*) and another between RS10685-10700 (including *pilV*, *pilW* and *pilX*) with two other orphan genes spread *pilW* (RS00345) and *pilT* (RS04490).

Searches for a T6SS identified three large gene clusters: RS05740-05865 was arranged in two operons, in opposing directions with a low %GC region between them; RS18005-18080 was arranged as one operon flanked by low %GC ratio regions; RS24120-24190 was arranged as one operon, except RS24190 in the opposing direction, flanked by low %GC ratio regions. Two single Hcp1 Type VI effector (RS18030 and RS24190) and VgrG (RS08970 and RS17060) were identified with high similarity to *P. fluorescens* (>90%).

In summary, all three genomes contained all or part of the three secretion systems. When considering the T3SS, the *P. tolaasii* 2192T NCPPB T3SS had similarity with T3SS of *P. agarici* NCPPB 2472 (RS18850-18985) and P. sp. NS1 (RS10335-10450). The T3SS of *Pseudomonas* sp. NS1 (RS06355-06465 and RS10335-10450) had similarity to T3SS of *P. agarici* NCPPB 2472 (RS25340-25440 and RS18850-18985, respectively) (Supplementary Material Table 5).

Table S1. Primers used in NV1 and ϕNV3 lysis protein constructs in this study.

Primer	Sequence
NV1HolinF	ATGGTATTCTTTGCTGCGTAC
NV1HolinR	TCATTTGGTTGCTCTCCATTC
NV1LysoF	ATGTCGCGGATCTCACTAC
NV1LysoR	TTATCCTTTATAAAGGTCGTACAGTG
Nv3aPin-F	ATGCAACTAGACACCACGAGCG
Nv3aPin-R	ATGTCGCGGATCTCACTACTGAGG
Nv3aRzl-F	ATGCGCTACGCCATCATTGCTG
3aLys_ex_R	ATCCAGCAATAGTGGCGAGTACG
3aPin_ex_R	TTCCTTAGGTTCATCCGCAGCC
3aRz1_ex_R	ATCCTCCAGTGGGTATCCATTAAGGG

Table S2. Table of all lysis protein constructs used in this study.

Construct	Protein
pEXP5-NV1Hol	Phage NV1 Holin
pEXP5-NV1Lys	Phage NV1 Lysozyme
pEXP5-NV3Pin	Phage ϕNV3 Pinholin
pEXP5-NV3Lys	Phage ϕNV3 Endolysin
pEXP5-NV3Rzl	Phage ϕNV3 Rzl/Rzl-like protein
PEXP5-NV3PinLys	Phage ϕNV3 Pinholin and Endolysin
pEXP5-NV3Full	Phage ϕNV3 Pinholin, Endolysin and Rzl/Rzl-like protein

Table S3. Plasmids used in this study.

Plasmid	Features	Source
pEXP5-CT/TOPO®	T7 promoter, T7 forward binding site, Ribosome binding site, TOPO® Cloning site, C-terminal polyhistidine (6xHis) tag, T7 transcription terminator, T7 reverse priming site, *bla* promoter, Ampicillin resistance gene (ß-lactamase), pUC origin of replication (*ori*)	Invitrogen, ThermoFisher Scientific, UK
pCR™2.1-TOPO®	LacZα fragment, M13 reverse priming site, Multiple cloning site, T7 promoter/priming site, M13 Forward (-20) priming site, f1 origin, Kanamycin resistance ORF, Ampicillin resistance ORF, pUC origin	Invitrogen, ThermoFisher Scientific, UK
pBBR1MCS-2	pBBR1 oriV, pBBR1 Rep, LacZα promoter, NeoR/KanR, M13 Forward priming site, M13 Reverse priming site, Multiple cloning site.	

Table S4. Predicted secondary metabolites and non-ribosomal peptide synthetase in *Pseudomonas tolaasii* 2192T, *P. agarici* NCPPB 2472 and *Pseudomonas* sp. NS1 genome.

P. tolaasii NCPPB 2192T	
**Locus tag**	**Predicted functions**
RS30620	non-ribosomal peptide synthetase
RS22935	non-ribosomal peptide synthetase
RS23980	non-ribosomal peptide synthetase
RS02650	non-ribosomal peptide synthetase
RS24725	non-ribosomal peptide synthetase
RS16160	non-ribosomal peptide synthetase
RS16165	pyoverdine synthesis
RS16170	pyoverdine synthesis
RS16175	TonB-dependent siderophore receptor
RS12420	siderophore synthetase
RS12390	siderophore synthetase
RS12395	MFS transporter
RS12400	TonB-dependent receptor
RS12430	TonB-dependent siderophore receptor
***P. agarici* NCPPB 2472**	
RS12910	non-ribosomal peptide synthetase
RS12915	non-ribosomal peptide synthetase
RS12920	non-ribosomal peptide synthetase
RS12925	non-ribosomal peptide synthetase
RS00065	non-ribosomal peptide synthetase
RS20800	non-ribosomal peptide synthetase
RS20810	non-ribosomal peptide synthetase
RS10630	non-ribosomal peptide synthetase
RS04660	RNA polymerase sigma factor
RS04665	sugar ABC transporter substrate-binding protein
RS04670	TonB-dependent siderophore receptor
RS04675	aspartate aminotransferase family protein
RS04680	AcsD protein
RS04685	diaminopimelate decarboxylase
RS04690	MFS transporter
RS04695	AcsC protein
RS04700	siderophore biosynthesis protein SbnG
RS04705	AcsA protein
***Pseudomonas* sp. NS1**	
RS19185	MATE family efflux transporter
RS19190	TetR/AcrR family transcriptional regulator
RS19195	MFS transporter
RS19200	toxin-antitoxin system HicB family antitoxin
RS19205	type II toxin-antitoxin system HicA family toxin

RS19210	non-ribosomal peptide synthetase
RS19215	peptide synthase
RS19220	non-ribosomal peptide synthetase
RS19225	non-ribosomal peptide synthetase
RS19230	non-ribosomal peptide synthetase
RS19235	TonB-dependent siderophore receptor
RS19240	cyclic peptide transporter
RS19245	N(5)-hydroxyornithine transformylase PvdF
RS19250	chromophore maturation protein PvdO
RS19255	aminotransferase
RS19260	peptidase M19
RS19265	PvdJ/PvdD/PvdP-like protein
RS19270	non-ribosomal peptide synthetase
RS19275	non-ribosomal peptide synthetase
RS19280	macrolide transporter subunit MacA
RS19285	MacB family efflux pump subunit
RS19290	LuxR family transcriptional regulator
RS19295	methionine gamma-lyase
RS19300	Lrp/AsnC family transcriptional regulator
RS26350	non-ribosomal peptide synthetase

Table S5. Predicted Type III (T3SS), IV (T4SS) and VI (T6SS) secretion systems in *Pseudomonas tolaasii* 2192T, *P. agarici* NCPPB 2472 and *Pseudomonas* sp. NS1 genome.

***P. tolaasii* NCPPB 2192T**	
**T3SS**	
**Locus tag**	**Predicted functions**
RS00310	tRNA-Gln
RS00335	sigma-54-dependent Fis family transcriptional
RS00340	hypothetical protein
RS00345	type III secretion system rspB
RS00350	EscJ/YscJ/HrcJ family type III secretion inner
RS00355	hypothetical protein
RS00360	type III secretion protein
RS00365	hypothetical protein
RS00370	hypothetical protein
RS00375	EscC/YscC/HrcC family type III secretion system
RS00380	type III secretion protein
RS00385	transcriptional regulator
RS00390	EscU/YscU/HrcU family type III secretion system export apparatus switch protein
RS00395	EscT/YscT/HrcT family type III secretion system export apparatus protein
RS00400	EscS/YscS/HrcS family type III secretion system export apparatus protein
RS00405	EscR/YscR/HrcR family type III secretion system export apparatus protein
RS00410	type III secretion protein
RS00415	flagellar hook-length control protein FliK
RS00420	type III secretion protein
RS00425	flagellum-specific ATP synthase FliI
RS00430	type III secretion protein
RS00435	EscV/YscV/HrcV family type III secretion system
RS00440	SepL/TyeA/HrpJ family type III secretion system gatekeeper
RS00445	RNA polymerase subunit sigma

RS00450	tRNA-Met
RS04585	EscC/YscC/HrcC family type III secretion system outer membrane ring protein
RS05820	hypothetical protein
RS05825	hypothetical protein
RS05830	hypothetical protein
RS05835	type III secretion protein
RS05840	EscV/YscV/HrcV family type III secretion systemexport apparatus protein
RS05845	type II and III secretion system protein RhcC2
RS05850	hypothetical protein
RS12630	translocation protein in type III secretionsystem, RhcU
RS12635	EscT/YscT/HrcT family type III secretion system export apparatus protein
RS12640	succinate dehydrogenase assembly factor 2 family protein
RS12645	hypothetical protein
RS12650	L-aspartate oxidase
RS12655	RNA polymerase sigma factor RpoE
RS12660	RNA polymerase subunit sigma
RS12665	RNA polymerase subunit sigma
RS12670	serine peptidase
RS12675	peptidase M48
RS12680	response regulator SirA
RS12685	EscC/YscC/HrcC family type III secretion system outer membrane ring protein
RS12690	peroxiredoxin
RSRS05495	type III effector
RSRS09075	type III effector HrpK
RSRS13030	type III secretion system effector protein ExoU
**T4SS**	
RS08725	pilus assembly protein
RS08730	pilus assembly protein PilX
RS08735	pilus assembly protein PilW
RS08740	type IV pilus modification protein PilV
RS08745	prepilin-type cleavage/methylationdomain-containing protein
RS10610	type IV pilus biogenesis/stability protein PilW
RS27580	pilus assembly protein
RS27585	pilus assembly protein
RS27590	Flp pilus assembly protein CpaB
**T6SS**	
RS14450	helicase
RS14455	type VI secretion-associated lipoprotein TagQ
RS14460	type VI secretion protein
RS14465	ABC transporter permease
RS14470	ABC transporter ATP-binding protein
RS26990	Hcp1 family type VI secretion system effector
RS16835	Hcp1 family type VI secretion system effector
RS25755	Hcp1 family type VI secretion system effector
RS14530	Hcp1 family type VI secretion system effector
RS18935	Hcp1 family type VI secretion system effector
RS05335	type VI secretion-associated protein
RS05340	type VI secretion system-associated protein
RS05345	type VI secretion system-associated protein
RS05350	type VI secretion protein
RS05355	hypothetical protein
RS05360	type VI secretion protein
RS05365	type VI secretion protein
RS05370	type VI secretion system-associated lipoprotein
RS05375	type VI secretion protein
RS25095	type VI secretion-associated protein
RS25100	type VI secretion protein
RS25105	hypothetical protein

RS25110	type VI secretion protein
RS25115	FHA domain-containing protein
RS25120	type VI secretion protein ImpA
RS25125	ClpV1 family T6SS ATPase
RS25130	type VI secretion protein
RS25135	type VI secretion system protein ImpG
RS25140	type VI secretion system lysozyme
RS25145	hypothetical protein
RS25150	EvpB family type VI secretion protein
RS25155	type VI secretion system-associated protein
RS25160	product=type VI secretion protein ImpA
RS25165	catalase
RS25170	anti-sigma factor
RS08870	type VI secretion protein VasK
RS25775	4-hydroxy-tetrahydrodipicolinate synthase
RS00795	type VI secretion protein VgrG
RS04750	type VI secretion protein VgrG
RS15065	type VI secretion protein VgrG
***P. agarici* NCPPB 2472**	
**T3SS**	
RS06355	hypothetical protein
RS06360	product=transcriptional regulator
RS06365	translocation protein in type III secretion system, RhcU
RS06370	type III secretion protein
RS06375	type III secretion protein
RS06380	type III secretion system protein SsaR
RS06385	hypothetical protein
RS06390	hypothetical protein
RS06395	type III secretion apparatus H+-transporting two-sector ATPase
RS06400	type III secretion protein
RS06405	hypothetical protein
RS06410	type III secretion protein
RS06415	hypothetical protein
RS06420	hypothetical protein
RS06425	type III secretion protein
RS06430	hypothetical protein
RS06435	hypothetical protein
RS06440	type II and III secretion system protein RhcC2
RS06445	type III secretion protein
RS06450	type III secretion protein
RS06455	hypothetical protein
RS06460	product=hypothetical protein
RS06465	hypothetical protein
RS22985	hypothetical protein
RS22990	product=D-alanyl-D-alanine carboxypeptidase
RS22995	nucleotide cyclase
RS23000	50S rRNA methyltransferase
RS23005	product=dihydroorotate dehydrogenase
RS23010	hypothetical protein
RS23015	invasion protein OrgB
RS23020	protein OrgA
RS23025	product=pathogenicity island 1 effector protein
RS23030	type III secretion system protein PrgJ
RS23035	product=protein MxiH
RS23040	type III secretion system protein PrgH
RS23045	invasion protein IagB
RS23050	invasion protein regulator

RS23055	acyl carrier protein
RS23060	hypothetical protein
RS23065	hypothetical protein
RS23070	pathogenicity island 1 effector protein SipB
RS23075	product=chaperone protein SicA
RS23080	type III secretion system protein SpaS
RS23085	product=type III secretion system protein SpaR
RS23090	type III secretion system protein SpaQ
RS23095	product=type III secretion system protein
RS23100	protein SpaO
RS23105	hypothetical protein
RS23110	protein SpaM
RS23115	ATP synthase
RS23120	Invasion protein B family
RS18850	type III effector HrpK
RS18855	RNA polymerase sigma factor RpoE
RS18860	type III secretion protein HrpJ
RS18865	Type III secretion inner membrane channel protein (LcrD,HrcV,EscV,SsaV)
RS18870	type III secretion protein HrpQ
RS18875	Type III secretion cytoplasmic ATP synthase (EC 3.6.3.14, YscN,SpaL,MxiB,HrcN,EscN)
RS18880	Type III secretion spans bacterial envelope protein (YscO)
RS18885	hypothetical protein
RS18890	hypothetical protein
RS18895	type III secretion protein HrcQb
RS18900	Type III secretion inner membrane protein (YscR,SpaR,HrcR,EscR,homologous to flagellar export components)
RS18905	Type III secretion inner membrane protein (YscS,homologous to flagellar export components)
RS18910	Type III secretion inner membrane protein (YscT,HrcT,SpaR,EscT,EpaR1,homologous to flagellar export components)
RS18915	Type III secretion inner membrane protein (YscU,SpaS,EscU,HrcU,SsaU, homologous to flagellar export components)
RS18920	hypothetical protein
RS18925	hypothetical protein
RS18930	negative regulator of hrp expression HrpV
RS18935	hypothetical protein
RS18940	Type III secretion outermembrane pore forming protein (YscC,MxiD,HrcC, InvG)
RS18945	type III secretion protein HrpG
RS18950	type III secretion protein HrpF
RS18955	hypothetical protein
RS18960	type III secretion protein HrpD
RS18965	Type III secretion bridge between inner and outermembrane lipoprotein (YscJ,HrcJ,EscJ, PscJ)
RS18970	type III secretion protein HrpB(Pto)
RS18975	hypothetical protein
RS18980	Type III helper protein HrpZ1
RS18985	type III helper protein HrpA
RS22995	nucleotide cyclase
RS23000	50S rRNA methyltransferase
RS23025	pathogenicity island 1 effector protein
RS23030	type III secretion system protein PrgJ
RS22985	hypothetical protein
RS03325	type III effector
RS18830	type III effector HrpK
RS07770	type III effector HopAC1
**T4SS**	
RS22640	signal peptidase II

RS22645	peptidylprolyl isomerase
RS22650	4-hydroxy-3-methylbut-2-enyl diphosphate reductase
RS22655	general secretion pathway protein GspH
RS22660	general secretion pathway protein GspH
RS22665	hypothetical protein
RS22670	pilus assembly protein PilW
RS22675	pilus assembly protein PilX
RS22680	pilus assembly protein
RS22685	pilus assembly protein
RS01575	chemotaxis protein CheW
RS01585	pilus assembly protein PilG
RS01635	type IV pili twitching motility protein PilT
RS06650	type IV pili twitching motility protein PilT
RS16265	type IV-A pilus assembly ATPase PilB
RS17195	pilus assembly protein PilW
**T6SS**	
RS02645	helicase
RS02650	type VI secretion-associated lipoprotein TagQ
RS02655	type VI secretion protein
RS02660	hypothetical protein
RS02665	ABC transporter ATP-binding protein
RS02670	serine/threonine protein kinase
RS02710	type VI secretion protein ImpA
RS02715	hypothetical protein
RS02720	EvpB family type VI secretion protein
RS02725	Hcp1 family type VI secretion system effector
RS02730	hypothetical protein
RS02735	hypothetical protein
RS02740	type VI secretion system lysozyme
RS02745	type VI secretion system protein ImpG
RS02750	type VI secretion protein
RS02755	ClpV1 family T6SS ATPase
RS02760	type VI secretion protein ImpA
RS02765	hypothetical protein
RS02770	hypothetical protein
RS21475	hypothetical protein
RS21480	hypothetical protein
RS21485	hypothetical protein
RS21490	resolvase
RS21495	transposase
RS21500	hypothetical protein
RS21505	integrase
RS21490	product=resolvase
RS21495	transposase
RS21500	product=hypothetical protein
RS21505	integrase
RS21510	product=ImpA-like protein
RS21515	product=fimbrial protein
RS21520	product=hypothetical protein
RS21525	type VI secretion system protein ImpM
RS21530	product=ImcF-like family protein
RS21535	product=type VI secretion system protein ImpK
RS21540	type VI secretion protein
RS21545	hypothetical protein
RS21550	ClpV1 family T6SS ATPase
RS21555	type VI secretion system protein
RS21560	product=type VI secretion system protein
RS21060	hydroxyacid dehydrogenase

RS21065	glucoamylase
RS21070	divalent metal cation transporter
RS21075	peptidyl-Asp metalloendopeptidase
RS21080	type VI secretion protein
RS21085	type VI secretion system protein ImpL
RS21090	type VI secretion system protein ImpK
RS21095	product=type VI secretion protein
RS21100	product=type VI secretion protein
RS21105	product=type VI secretion protein
RS21110	product=EvpB family type VI secretion protein
RS21115	type VI secretion effector protein (Hcp)
RS21120	type VI secretion protein
RS21125	product=type VI secretion system protein ImpG
RS21130	type VI secretion protein
RS21135	ClpV1 family T6SS ATPase
RS12550	type VI secretion protein
RS12555	EvpB family type VI secretion protein
RS12560	type VI secretion protein
RS12565	type IV secretion protein DotU
RS12570	flagellar motor protein MotB
RS12575	Hcp family T6SS protein CtsH1
RS12580	ClpV1 family T6SS ATPase
RS12585	type IV secretion protein Rhs
RS09100	type VI secretion system lysozyme-like protein
RS19520	Hcp1 family type VI secretion system effector
RS19150	Hcp1 family type VI secretion system effector
RS09880	Hcp1 family type VI secretion system effector
RS02725	Hcp1 family type VI secretion system effector
RS01160	Hcp1 family type VI secretion system effector
RS01355	type IV secretion protein Rhs
RS05175	type IV secretion protein Rhs
RS13620	type IV secretion protein Rhs
RS20175	type IV secretion protein Rhs
***Pseudomonas* sp. NS1**	
**T3SS**	
RS10335	RNA polymerase sigma-54 factor RpoN
RS10340	hypothetical protein
RS10345	Type III secretion inner membrane channel protein (LcrD,HrcV,EscV,SsaV)
RS10350	hypothetical protein
RS10355	type III secretion protein HrpQ
RS10360	Type III secretion cytoplasmic ATP synthase (EC 3.6.3.14, YscN,SpaL,MxiB,HrcN,EscN)
RS10365	hypothetical protein
RS10370	hypothetical protein
RS10375	hypothetical protein
RS10380	Type III secretion inner membrane protein (YscR,SpaR,HrcR,EscR,homologous to flagellar export components)
RS10385	Type III secretion inner membrane protein (YscS,homologous to flagellar export components)
RS10390	Type III secretion inner membrane protein (YscT,HrcT,SpaR,EscT,EpaR1,homologous to flagellar export components)
RS10395	Type III secretion inner membrane protein (YscU,SpaS,EscU,HrcU,SsaU, homologous to flagellar export components)
RS10400	negative regulator of hrp expression HrpV
RS10405	type III secretion protein HrpT
RS10410	Type III secretion outermembrane pore forming protein (YscC,MxiD,HrcC, InvG)
RS10415	type III secretion protein HrpG

RS10420	hypothetical protein
RS10425	Type III secretion cytoplasmic protein (YscL)
RS10430	type III secretion protein HrpD
RS10435	Type III secretion bridge between inner and outermembrane lipoprotein (YscJ,HrcJ,EscJ, PscJ)
RS10440	hypothetical protein
RS10445	hypothetical protein
RS10450	Pathogenicity locus probable regulatory protein hrpR
RS25340	hypothetical protein
RS25345	Type III secretion inner membrane protein (YscU,SpaS,EscU,HrcU,SsaU, homologous to flagellar export components)
RS25350	Type III secretion inner membrane protein (YscT,HrcT,SpaR,EscT,EpaR1,homologous to flagellar export components)
RS25355	Type III secretion inner membrane protein (YscS,homologous to flagellar export components)
RS25360	Type III secretion inner membrane protein (YscR,SpaR,HrcR,EscR,homologous to flagellar export components)
RS25365	hypothetical protein
RS25370	hypothetical protein
RS25375	Type III secretion cytoplasmic ATP synthase (EC 3.6.3.14, YscN,SpaL,MxiB,HrcN,EscN)
RS25380	Type III secretion cytoplasmic protein (YscL)
RS25385	hypothetical protein
RS25390	Type III secretion bridge between inner and outermembrane lipoprotein (YscJ,HrcJ,EscJ, PscJ)
RS25395	hypothetical protein
RS25400	hypothetical protein
RS25405	Type III secretion outermembrane pore forming protein (YscC,MxiD,HrcC, InvG)
RS25410	hypothetical protein
RS25415	hypothetical protein
RS25420	Type III secretion outermembrane contact sensing protein (YopN,Yop4b,LcrE)
RS25425	Type III secretion inner membrane channel protein (LcrD,HrcV,EscV,SsaV)
RS25430	hypothetical protein
RS25435	hypothetical protein
RS25440	hypothetical protein
RS29245	EscC/YscC/HrcC family type III secretion system outer membrane ring protein
RS00750	type III effector
RS04255	type III secretion system effector protein exou
**T4SS**	
RS08735	Type IV pilus biogenesis protein PilM
RS08745	Type IV pilus biogenesis protein PilP
RS08750	Type IV pilus biogenesis protein PilQ
RS10685	Type IV fimbrial biogenesis protein PilV
RS10690	Type IV fimbrial biogenesis protein PilW
RS10695	Type IV fimbrial biogenesis protein PilX
RS10700	Type IV pilus biogenesis protein PilE
RS00345	Type IV pilus biogenesis protein PilF
RS04490	twitching motility protein PilT
**T6SS**	
RS05740	hypothetical protein
RS05745	Autotransporter adhesin
RS05750	hypothetical protein
RS05755	ABC-type antimicrobial peptide transport system, permease component
RS05760	ABC transporter, ATP-binding protein
RS05765	T6SS Serine/threonine protein kinase (EC 2.7.11.1) PpkA
RS05770	T6SS protein serine/threonine phosphatase PppA
RS05775	T6SS associated component TagF (ImpM)
RS05780	T6SS component TssM (IcmF/VasK)

RS05785	T6SS outer membrane component TssL (ImpK/VasF) / OmpA/MotB domain
RS05790	T6SS component TssK (ImpJ/VasE)
RS05795	Type VI secretion lipoprotein/VasD
RS05800	T6SS forkhead associated domain protein ImpI/VasC
RS05805	T6SS component TssA (ImpA)
RS05810	T6SS component TssB (ImpB/VipA)
RS05815	T6SS component TssC (ImpC/VipB)
RS05820	T6SS component Hcp
RS05825	T6SS lysozyme-like component TssE
RS05830	T6SS component TssF (ImpG/VasA)
RS05835	T6SS component TssG (ImpH/VasB)
RS05840	T6SS AAA+ chaperone ClpV (TssH)
RS05845	ClpV1 family T6SS ATPase
RS05850	FIG00964469: hypothetical protein
RS05855	hypothetical protein
RS05860	hypothetical protein
RS05865	putative transmembrane anti-sigma factor
RS18010	Catalase-like heme-binding protein
RS18020	T6SS component TssB (ImpB/VipA)
RS18025	T6SS component TssC (ImpC/VipB)
RS18030	T6SS component Hcp
RS18035	T6SS lysozyme-like component TssE
RS18040	T6SS component TssF (ImpG/VasA)
RS18045	T6SS component TssG (ImpH/VasB)
RS18050	T6SS AAA+ chaperone ClpV (TssH)
RS18055	type VI secretion protein ImpA
RS18060	hypothetical protein
RS18065	T6SS component TssK (ImpJ/VasE)
RS18070	T6SS outer membrane component TssL (ImpK/VasF) / OmpA/MotB domain
RS18075	T6SS component TssM (IcmF/VasK)
RS18080	T6SS associated component TagF (ImpM)
RS24120	T6SS component TssM (IcmF/VasK)
RS24125	T6SS outer membrane component TssL (ImpK/VasF)
RS24130	T6SS component TssK (ImpJ/VasE)
RS24135	T6SS secretion lipoprotein TssJ (VasD)
RS24140	type VI secretion protein
RS24145	type VI secretion protein
RS24150	T6SS sigma-54-dependent regulator VasH
RS24155	T6SS AAA+ chaperone ClpV (TssH)
RS24160	T6SS component TssG (ImpH/VasB)
RS24165	T6SS component TssF (ImpG/VasA)
RS24170	T6SS lysozyme-like component TssE
RS24175	T6SS component TssC (ImpC/VipB)
RS24180	T6SS component TssB (ImpB/VipA)
RS24185	hypothetical protein
RS24190	T6SS component Hcp
RS08970	type IV secretion protein Rhs
RS17060	type IV secretion protein Rhs

Table S6. Predicted prophage in *Pseudomonas tolaasii* 2192T, *P. agarici* NCPPB 2472 and *Pseudomonas* sp. NS1 genome.

***P. tolaasii* NCPPB 2192T**				
**Completeness**	**Region Length**	**Total Proteins**	**Region Position (bp)**	**GC %**
intact	33.5Kb	51	496186-529725	57.67%
intact	52.1Kb	58	4609424-4661540	57.75%
intact	32.1Kb	26	4901329-4933507	60.61%
intact	44Kb	53	5295871-5339955	58.18%

intact	54.6Kb	76	6763248-6817888	52.42%
incomplete	17.6Kb	21	885369-903001	57.49%
incomplete	16.8Kb	17	1400028-1416864	54.14%
incomplete	26.9Kb	21	1749306-1776277	57.84%
questionable	17.5Kb	20	3517221-3534763	60.41%
***P. agarici* NCPPB 2472**				
incomplete	22.5Kb	22	2495393-2517980	58.84%
incomplete	17.1Kb	13	2791355-2808524	58.56%
***Pseudomonas* sp. NS1**				
intact	46.2Kb	48	3132347-3178632	54.77%
incomplete	25.4Kb	23	710706-736117	56.28%
incomplete	21.6Kb	29	1693449-1715135	56.95%
incomplete	25.3Kb	8	4965531-4990894	59.15%
incomplete	8.5Kb	8	6232104-6240655	52.13%

Table S7. Predicted phage tail-like bacteriocin in Pseudomonas tolaasii 2192T, P. agarici NCPPB 2472 genome.

**P. tolaasii NCPPB 2192T**	
**Locus tag**	**Predicted functions**
RS06590	Pyocin activator protein PrtN
RS16305	transcriptional regulator
RS16310	pyocin R2, holin
RS16315	hypothetical protein
RS16320	phage baseplate protein assembly
RS16325	phage baseplate protein
RS16330	baseplate J protein
RS16335	phage tail protein I
RS16340	tail fiber protein
RS16345	hypothetical protein
RS16350	hypothetical protein
RS16355	tail protein
RS16360	phage tail protein
***P. agarici* NCPPB 2472**	
RS10915	pyocin R2, holin
RS10920	hypothetical protein
RS10925	DUF2635 domain-containing protein
RS10930	phage tail protein
RS10935	tail protein
RS10940	hypothetical protein
RS10945	phage tail protein
RS10950	hydroxyacid dehydrogenase
RS10955	baseplate protein
RS10960	hypothetical protein
RS10965	hypothetical protein
RS10970	baseplate J protein
RS10975	phage tail protein
RS10980	hypothetical protein
RS10985	acyltransferase
RS10990	pyocin R, lytic enzyme
RS10995	lysozyme
RS18570	pyocin activator protein PrtN

Table S8. Predicted phage resistance systems in Pseudomonas tolaasii 2192T, P. agarici NCPPB 2472 and Pseudomonas sp. NS1 genome.

***P. tolaasii* NCPPB 2192T**	
**Locus tag**	**Predicted functions**
RS17360	Restriction endonuclease
RS17410	Restriction endonuclease subunit R
RS26390	Restriction endonuclease subunit M
RS30090	Restriction endonuclease subunit M
RS30095	Restriction endonuclease subunit M
RS11470	Short-chain dehydrogenase
RS11475	Hypothetical protein
RS11480	DUF3077 domain-containing protein
RS11485	Mannose-1-phosphate guanylyltransferase/mannose-6-Phosphate isomerase
RS11490	Alginate O-acetyltransferase
RS11495	Alginate O-acetyltransferase
RS11500	MBOAT family protein
RS11505	Mannuronate-specific alginate lyase
RS11510	Alginate O-acetyltransferase
RS11515	Right-handed parallel beta-helix repeat-containing protein
RS11520	Alginate biosynthesis protein AlgE

RS11525	Sel1 repeat family protein
RS11530	Alginate biosynthesis protein Alg44
RS11540	Glycosyl transferase
RS11545	Nucleotide sugar dehydrogenase
***P. agarici* NCPPB 2472**	
RS05180	Type I-F CRISPR-associated endonuclease Cas1
RS05185	Type I-F CRISPR-associated helicase Cas3
RS05190	Type I-F CRISPR-associated protein Csy1
RS05195	Type I-F CRISPR-associated protein Csy2
RS05200	Type I-F CRISPR-associated protein Cas7f/Csy3
RS05205	Type I-F CRISPR-associated endoribonuclease Cas6/Csy4
RS05570	Restriction endonuclease
RS05645	Restriction endonuclease subunit R
RS18565	Restriction endonuclease
RS18570	Type I restriction endonuclease subunit R
RS24540	Restriction endonuclease subunit S
RS18660	Restriction endonuclease subunit S
RS18670	Type I restriction endonuclease subunit R
***Pseudomonas* sp. NS1**	
RS15035	restriction endonuclease subunit M
RS21265	restriction endonuclease subunit S
RS27500	restriction endonuclease (McrC)
RS27505	restriction endonuclease (McrB)

Figures

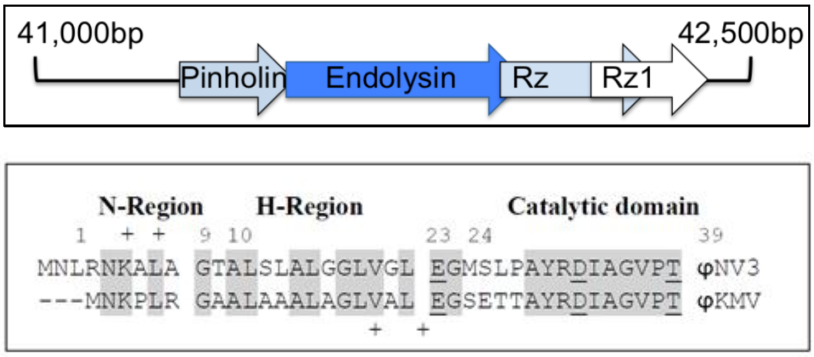

Figure S1. lysis cassette of phage ϕNV3, top box shows the ORF's encoding the putative pinholin, endolysin, Rz and Rz1-like proteins and the high degree of overlap between each ORF's; The bottom box is the direct comparison of the N-terminal *signal-arrest-release* domains of ϕNV3 and ϕKMV. The positively charged N-region, the hydrophobic H-region and the catalytic domain are indicated with the conserved catalytic domains underlined.

References

1. Jackson, R. W.; Preston, G. M.; Rainey, P. B., Genetic characterization of *Pseudomonas fluorescens* SBW25 rsp gene expression in the phytosphere and in vitro. *J Bacteriol* **2005,** *187*, (24), 8477-88.
2. Preston, G. M.; Bertrand, N.; Rainey, P. B., Type III secretion in plant growth-promoting *Pseudomonas fluorescens* SBW25. *Molecular microbiology* **2001,** *41*, (5), 999-1014.
